# Dissociation in mothers with borderline personality disorder: a possible mechanism for transmission of intergenerational trauma? A scoping review

**DOI:** 10.1186/s40479-024-00250-7

**Published:** 2024-03-11

**Authors:** David Rimmington, Rachel Roberts, Alyssa Sawyer, Anne Sved-Williams

**Affiliations:** 1 School of Psychology, The University of Adelaide, North Terrace, Adelaide, SA Australia; 2 Perinatal and Infant Mental Health Services, Women’s and Children’s Hospital, North Adelaide, SA Australia; 3 Australian National University, ACT, Canberra, SA Australia

**Keywords:** Dissociation, Perinatal, Mothers, Borderline, Attachment, Trauma

## Abstract

**Background:**

Dissociation is a feature of Borderline Personality Disorder (BPD), but rarely a focus for research, particularly in the perinatal literature. BPD partly has its aetiology in childhood and is characterised by emotional changes and difficulty with self-coherence that impacts on the processes of caregiving.

**Methods:**

A scoping review was conducted to synthesise current perspectives on the effect of dissociation in caregivers with BPD, particularly regarding the impact of caregiver dissociation on the interactional quality of relationship within parent–child dyads. Studies were included if they explicitly mentioned dissociation in the target population, or if dissociation was implied. A thematic analysis was conducted.

**Results:**

20 studies were included; 10 experimental or quasi-experimental; 2 presenting case material; and 8 non-systematic review articles. 4 studies used the Dissociative Experiences Scale (DES) to measure dissociation, while 2 studies included a ‘dissociative behaviour’ subscale as part of an observational measure. The remaining studies did not measure dissociation but referenced directly or indirectly a concept of dissociation.

**Conclusions:**

Findings suggested there was some evidence that dissociation plays a unique role in BPD caregivers’ interactions with their offspring, however any findings should be interpreted with caution as the concept has been poorly operationalised and defined.

**Supplementary Information:**

The online version contains supplementary material available at 10.1186/s40479-024-00250-7.

## Background

It is well known that during development, quality of caregiving can exert influence on a variety of physical health and neurological conditions [1–5]. An important factor in healthy caregiving is the psychological makeup of the caregiver, which can contribute to the ability to provide care to an infant or child [6]. Caregivers wish to provide adequate care, however caregiving resources both internal (such as patience, emotion regulation capabilities and reflective functioning) and external (time, money, social support) are not distributed equally. There is a growing interest in screening for caregiver mental health issues including perinatal anxiety and depression [7], and between 10 – 22% of parents experience a mental disorder in a western context [8–10]. Where parents face challenges due to mental health disorders children are also reported to experience poorer outcomes including higher rates of mental illness [11] as well as higher rates of neurodevelopmental issues [12], and functional impairment in schooling and academic achievement [13]. Despite the high rates of exploration of caregiver perinatal depression and anxiety on children, there is less research on borderline personality disorder (BPD) in the caregiving context.

### Borderline personality disorder and caregiving

BPD affects roughly 1–6% of the population [14] with an over representation in psychiatric contexts at around 10–15% [15]. Rates of BPD in caregivers in a clinical context are estimated to be around 14% [16]. Those with BPD are more likely to experience a host of negative outcomes including self-harm [17], and death by suicide [18], as well as lower quality of life [19] and relationship difficulties [20].

To date, limited evidence however has examined the specific effects of BPD on parenting practices. There is some evidence to suggest BPD mothers may experience difficulty providing care to distressed infants as the duration of infant distress increases [21]. Research suggests also that mothers with BPD may experience particular subjective difficulty in response to persistent crying from their infants [22].

Although caregiver emotional dysregulation and inconsistent responding may occur, there is a lack of understanding of putative mechanisms behind disordered caregiver-infant interactions. Eyden et al. [23] note “precisely how parenting strategies unravel between mother-infant dyads requires further explication” (p. 102). Efforts to explicate underlying interactional mechanisms that contribute to disrupted caregiving in this group are particularly important when considering that there is no difference in reported caregiver concern for children in this group [24–26] as compared to other populations. BPD caregivers wish to care for their children, but due to these mechanisms this caregiving ability may be undermined.

### Role of dissociation in BPD parents

A mechanism that has been suggested as leading to inconsistent caregiving is dissociation, or dissociative caregiving [27]. Dissociation can be defined broadly as disruption in subjective experience in a psychological system [28]. It can be pathological but also non-clinical [29] and acute (state dissociation) or chronic (trait dissociation). Dissociation is seen as important in the context of BPD in particular, with a recent review of neural imaging studies suggesting the importance of dissociation in a variety of symptoms and outcomes [30].

Looking at functional neurological research into dissociation in BPD, there is evidence that when dissociation is induced, people with BPD appear less able to inhibit negative emotional responses and appear to find it harder to focus on non-aversive stimuli [31]. The mechanism of dissociation is possibly disruptive in those with BPD in their attempts to deploy positive parenting behaviours. In general, however, the literature on dissociation and in particular in dissociative caregiving is scant. Dissociation as a phenomenological construct in children has been linked to poor developmental progress [32]. Dissociation in mothers has also been found to predict dissociation in children [33]. In adult populations, dissociation as a symptom in BPD has been associated with more suicidal or self-injurious behaviour, and therefore is proposed to be an important target for screening and intervention [34]. Dissociation in caregivers has also been studied in mothers with post-traumatic stress disorder (PTSD), where functional magnetic resonance imaging (fMRI) has been applied to image mothers with subjectively high reports of dissociative symptoms whilst observing video of separation events of children [35]. Findings suggest excessive inhibitory activity present in traumatised populations which in turn is suggestive of downregulation in emotional sensitivity and perhaps attentional awareness. Given the current findings, it would appear that dissociation in caregivers is possibly a viable target for intervention and implicated in significant psychopathology in adult populations and also in caregiving responses. However, there is still a large gap in the literature regarding the impact of dissociation in caregivers with BPD.

### Theories of dissociation in BPD

Dissociation in the BPD population, whilst a possible mechanism of concern, has been difficult to conceptualise and study [36]. One reason for the difficulty is related to the variable definitions and importance placed on dissociation in this context.

Some theorists point to dissociation as being fundamental to BPD, and occurring on three different ‘levels’ (primary, secondary, and tertiary), all causing various difficulties and encompassing different phenomena [37]. Other theorists hold similar views but suggest a primary and secondary dissociation as being related to defensive processes and (subsequent) division within the personality, which leads to long term difficulty in integrating emotional experiences into everyday personality functioning [38]. Within this framework, BPD is one of a spectrum of disorders that can occur depending on factors related to severity and chronicity of personality ‘divisions’. Elsewhere, some accounts suggest that BPD may have roots in disorganised patterns of attachment. Specifically, parents who display so-called ‘frightened / frightening’ behaviours [39] are thought to contribute to infant disorganisation when the mother displays them to the infant. These behaviours are possibly dissociative behaviours, representing dissociative intrapsychic processes in the parent. BPD parents may display these behaviours to their offspring, re-activated by the caregiving situation [39]. Further elucidation is offered by Amos, Furber and Segal [40] who present an integrative model of dissociation, trauma, and attachment theory which the authors use to offer theoretical explanation for the maltreatment of children. In their work, dissociation is proposed as the mechanism that enables a shift from what might be ‘adaptive’ parenting into maladaptive, or abusive, parenting.

Elsewhere in adult literature, some researchers suggest that the majority of persons with BPD may have a dissociative disorder [41]. Further adding to conceptual confusion is contention between perspectives on the aetiology of dissociation. Two dominant perspectives are evidenced, one suggesting that dissociation is ‘trauma based’ and directly related to experiencing trauma and the other ‘socio-cognitive’ explanation suggesting dissociation is socio-culturally influenced (for a review see [42]).

Some researchers suggest that dissociation causes reduced treatment effects across disorders [43], and in a BPD context there is evidence that persons who experience high levels of dissociation are less amenable to general BPD treatment [44]. Treatment approaches for those with BPD tend to address dissociation either passively or actively depending on orientation, however, only recently have guidelines been developed for treatment of dissociation in BPD populations [45]. The difficulty in treatment and different perspectives on dissociation in BPD results in methodological difficulties in relation to assessing and understanding the effect of dissociation on BPD caregivers. Specifically, issues such as different trauma histories, murky boundaries between diagnoses, and unclear definition of dissociation has been suggested to lead to problems in understanding and addressing dissociation in the BPD population [46].

### Dissociation and intergenerational trauma

The following illustrative account of dissociation and its influence on intergenerational transmission of trauma can be provided, based on disparate research 1) Disorganised attachment in childhood is highly correlated with adult experience of dissociation [47, 48], 2) BPD is correlated with experience of disorganised attachment [49], 3) it follows that there is perhaps a correlation between dissociative symptoms and BPD through the experience of ‘disorganised attachment’ (which can itself be conceptualised as a dissociative phenomenon [4]). The caregiving context may present a neurobiologically ‘primed’ situation in which caregivers may (re-)experience dissociative phenomena, in relation to their offspring, 5) this may be impossible to report accurately (subjectively) due to the nature of the experienced phenomena, thus impairing caregivers’ ability to seek and benefit from intervention 6) children may be impacted by dissociative caregiving in a unique way due to the behaviour putting the child in a state of ‘fright without solution’ [39] as there is no primary caregiver to rely upon for safety. Where the caregiver is the cause of fear also, a uniquely stressful experience for the child is created [7] the child’s physiological response of heightened emotional activation (attempts to elicit soothing behaviour) may provoke dissociative experiences in the caregiver, which in BPD can manifest as maltreating or seriously disruptive behaviours [8]. Caregiver dissociation therefore becomes the mechanism which is relied upon unconsciously to protect the caregiver from overwhelming experience, simultaneously disabling helpful repair that would help regulate the child and possibly the caregiver. Dissociation is therefore seen as important in the transmission of intergenerational trauma. Studies have shown that maternal dissociation is associated with children’s dissociation and trauma symptoms [50] in other populations. However, due to a lack of coherent perspectives and the methodological issues, empirical validation of the mechanisms of transmission and the BPD context remains unclear.

### Aims of the study

Given the lack of clarity around the nature of caregiver dissociation in this population, and the theoretical importance of such a construct, a scoping review was conducted to assess the current state of the literature and identify the need or otherwise for future research in the area.

This study sought to identify, evaluate, and synthesise the existing literature regarding dissociation in caregivers with BPD, and the impacts of caregiver dissociation on children Specifically, the following were investigated:

1. What theoretical models of dissociation are being used to examine parenting with BPD?
2. What work has been done to understand the role of dissociation in this group of caregivers?
3. What evidence is there regarding the impact of intervention on dissociation in caregivers with BPD?
4. How does dissociation relate to the transmission of intergenerational psychological trauma (from caregiver to child)?

## Methods

### Framework and registration

This scoping review used the Population, Concept, and Context (PCC) framework recommended by Joanna Briggs Institute for scoping reviews [51] to frame research questions and eligibility criteria. The protocol for this scoping review was pre-registered on Open Science Framework (10.17605/OSF.IO/V5SHC) using their tool and framework, which promotes transparent sharing of the research process. The Preferred Reporting Items in Systematic Reviews and Meta-analyses extension guidelines for scoping reviews (PRISMA-ScR; [52]) were followed and adhered too; a full checklist can be found in supplementary material (Supplement 1).

### Search strategy

The search strategy adopted was broadly three phases. The first phase was a preliminary search of MEDLINE, the Cochrane database of systematic reviews and *JBI Evidence Synthesis,* to determine that no current or underway systematic reviews or scoping reviews on the topic of dissociation within a parenting context in those with BPD were identified. Second, an initial limited search was then undertaken to identify articles relevant to the topic to generate more accurate keywords for the comprehensive search. Key terms, deriving from broadly [dissociation AND caregiving / parents AND Borderline Personality Disorder] were decided upon through this process; the exact keywords were made database specific by utilizing the databases’ thesauri (see supplementary material for full search terms). A research librarian was consulted to ensure comprehensiveness and the inclusion of grey literature and unpublished studies. Finally, a search of ten databases was then conducted (PubMed, PsycINFO, PsychEXTRA, PubMed Central, PsycArticles, Embase, CINAHL, Scopus, Web of Science, PTSDPubs). The search strategy, including databases utilised and keywords, was deliberately as broad as possible given then specific nature of the research questions. In other words, the researchers wanted to capture as much as possible due to the conceptual ‘layers’ (borderline, parenting, dissociation), and the likelihood that dissociation would be an area of relatively little empirical research but implicated in many theoretical discussions, and perhaps addressed through other terms. The initial searches were run on 24/07/2021, 29/07/2021 and 30/07/2021 and results were imported to the Covidence software (www.covidence.org) for compilation, screening, exclusion, and extraction. Searches were set to automatically update and email the first author new results each month until data were analysed, and screening completed (20/11/2022; *n* = 32) and continued until analysis and synthesis was completed on 24/06/2023 (*n* = 20). Logic grids with different searches being built for database specific terms are provided in supplementary material (Supplement 2).

### Inclusion criteria

The review adopted an iterative approach to included studies. This approach was chosen to dynamically screen in and out references based on updated findings. Because of the expected theoretical nature of the constructs chosen (as opposed to expecting predominantly quantitative studies), it was important to ensure that the search strategy could be updated, and articles could be included or excluded as the authors’ gained understanding of the constructs. Sources were included if they 1) mentioned caregiving, dissociation, and BPD, 2) focussed on caregiver interactions or caregiver pathology rather than offspring outcomes. Studies that mentioned BPD but did not focus exclusively on the diagnosis were included. Any studies that mentioned dissociation were initially included. Studies that mentioned constructs indicating dissociative phenomena (specifically, depersonalisation, derealisation, freeze responses, hallucinations) either in the theoretical considerations and explanatory (interpretive) hypotheses presented in discussion, or where a more explicit mention or measurement of caregiver dissociation was made, were included. Studies that mentioned constructs associated with so called non-pathological dissociation, specifically daydreaming and absorption were included for review also, under the rationale that these behaviours may constitute dissociation that could be problematic or defensive in a relational context. In iterative considerations of inclusion criteria, the decision was made to include any studies that utilised measures of caregiver interaction where coding of interactions was suggestive of dissociative behaviours, and raw data was provided. The rationale behind this was the frequent reference to “Hostile / Helpless” caregiving [53], where the original article based the “Hostile / Helpless” paradigm on dissociated Internal Working Models. A full explication of the intricacies of these theories is beyond the scope of the current article; however, measures assessing or including Hostile / Helpless constructs (primarily AMBIANCE [54]) were determined to be behavioural or phenomenological measures of ‘dissociation’ in part, and thus were included. This decision was made to assess completely the relevant literature where dissociation may not have been an explicit focus of discussion, but nonetheless may have been measured in some capacity in the population. Grey literature including case studies, trials, theoretical articles were all included.

### Study selection

As above, given the authors’ contention that ‘dissociation’ is a construct of significant heterogeneity, the definition of dissociation was broadened for the study. A priori and a posteriori definitions were therefore accepted as were studies that indicated dissociation rather than explicitly writing about it. In compiling a systematic mapping of the literature, the aim was to collate all related information and synthesise it.

**Fig. 1:**
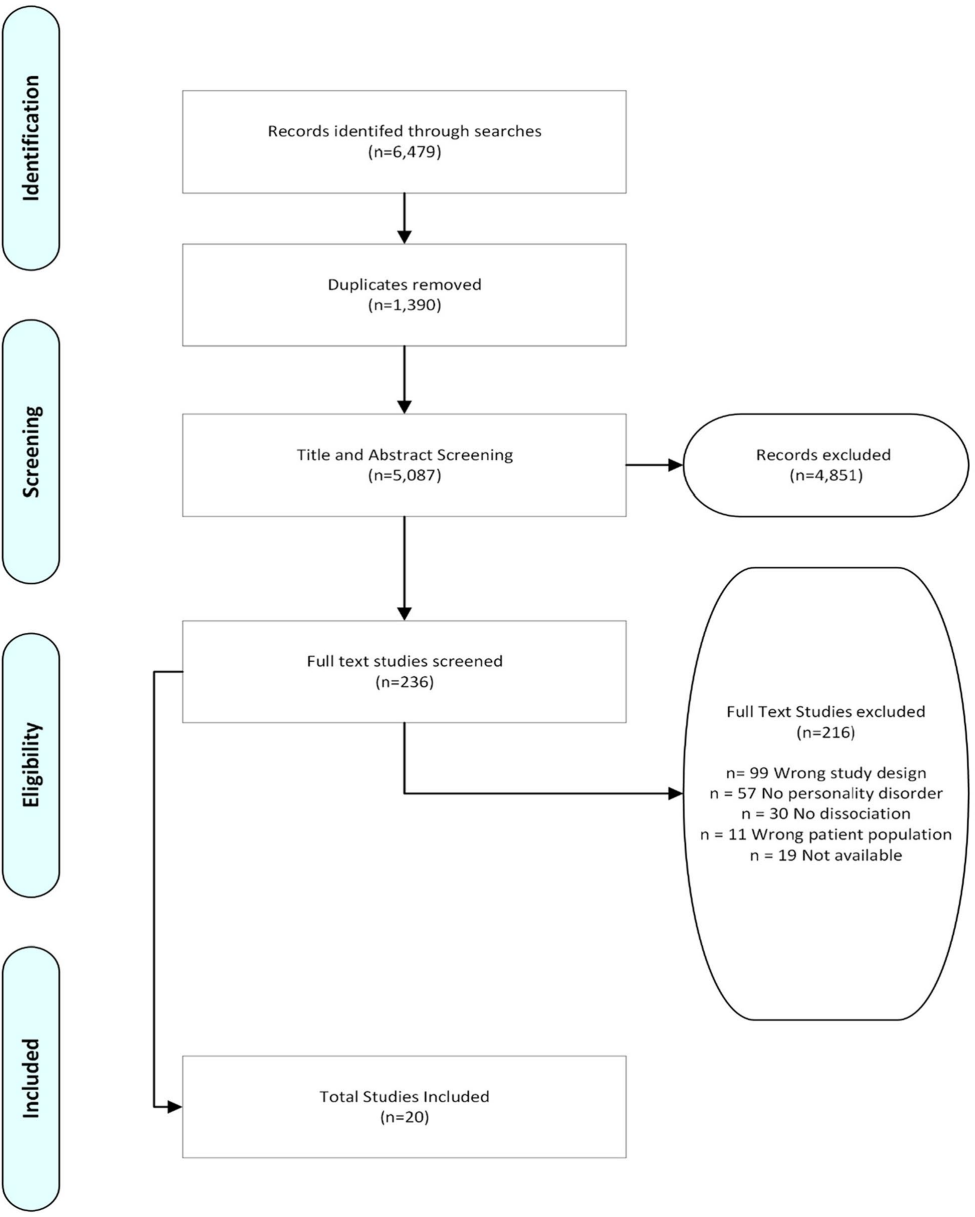
PRISMA Flowchart of study selection process

A subset of the data was screened by two reviewers at the title and abstract and full text eligibility stages, to limit data selection bias and determine inter-rater reliability. At the title and abstract stage *n* = 50 records were co-screened. Good inter-rater reliability was found (k = 0.96) and disagreements were discussed, and all resolved. At the full-text stage, a document regarding inclusion criteria was developed to improve inter-rater reliability. A random set of the records was co-screened (*n* = 20). Proportionate agreement was found in 85% of records (*n* = 17). Disagreements were resolved through discussion and as such no further reviewer was necessary. The screening process is detailed in the PRISMA flow chart (Fig. 1). Two hundred thirty-six (*n* = 236) studies were retained for full-text screening, with two hundred sixteen (*n* = 216) excluded at this stage and a total of twenty (*n* = 20) articles retained for extraction. All studies that were selected for full text extraction bar two book chapters, were journal articles; none were grey literature or governmental reports. Reasons for exclusion at the full text stage are detailed in Fig. 1 below.

### Data extraction

The method of scoping review as outlined by [51] was used as the analytic framework. Therefore, the review organised data into a data charting table. The data charting table is presented in full in supplementary material (Supplement 3); a truncated version of the table is presented below (Table 1). The included studies adopted varied methodologies and reported on different aspects of caregiver-child relations; some articles were theoretical, some mixed methods. Given the heterogeneity of included studies, data was extracted where applicable using the data charting table, but results were not excluded if data was not present. For instance, qualitative studies that focussed on theory were expected to have a theoretical focus and thus provide descriptions of dissociation and theories of the construct but were not excluded if they did not include measures of dissociation. Extracted data included the reference, study design, study aims, population, measures and methodology, and a brief review of relevant findings, authors conclusions and potential study limitations. The charting table documentation adopted an iterative approach, as per the overall goals of a scoping review, as it was not clear initially what data would be found. The iterative building of the data charting table allowed for flexible revision and thus to adaptively understand the material presented, building a more complete map of the relevant research.

**Table 1:** Summary of included sources (descriptive information)

1st Author (Year), Location, Type	Design	Aims	Population	Methods	What work has been done to advance understanding of dissociative caregiving in this population?
Blizard (2003), USA, Journal Article [55]	Non-systematic review and opinion	Not explicitly stated. To present a theoretical model of disorganized attachment and dissociation, and a treatment model based on findings	N/A	Theoretical discussion and synthesis and presentation of case material	Theoretical discussion with explicit theoretical basis
Crandell (2003), UK, Journal Article [56]	Non-randomised experimental study	To investigate mother-infant relations when mothers have borderline personality disorder	Mothers with borderline personality disorder + their infants; control group of mothers with no history of disorder + their infants	No dissociation measures. Videotape analysis and rating by blinded rater based on previous study criteria. Measured pre- and post-still-face procedure, and during face-to-face play post-still-face	Observation of behaviour with implicit idea
Haltigan (2019), Canada, Journal Article [57]	Latent trait modelling analysis of AMBIANCE measure (factor analysis)	To apply the Item Response Theory (IRT) modelling techniques to the Atypical Maternal Behaviour Instrument for Assessment and Clarification (AMBIANCE)	Item-level AMBIANCE data; 6 subsamples (pooled *n* = 343) from various parent studies conducted in western countries	Item level data on the AMBIANCE measure pooled; transformed to binary scale and analysed	Observation of behaviour with explicit focus or mention
Hesse (2000) USA, Journal Article [39]	Non-systematic review and opinion	Provide a descriptive account of processes that identify disorganized attachment status across lifespan	Presentation of cases and summary synthesis of literature regarding disorganised attachment	Presentation and synthesis of ideas relating to attachment, disorganised attachment, and dissociation	Observation of behaviour with explicit focus or mention; Theoretical discussion where there is no explicated model
Hobson (2005), UK, Journal Article [58]	Non-randomised experimental study	Assessment of interactional quality and attachment of 12-month-old infants of mothers with BPD; evaluation of maternal intrusive insensitivity	Mother infant Dyads with 12-month old infants. 10 mothers with BPD; 22 control mothers without psychopathology	Modified set situation was applied to each mother infant dyad. Still face phase (90 s). Rapprochement and spatula phase then administered. Rating of whole situation then applied	Observation of behaviour with implicit idea
Hobson (2009), UK, Journal Article [59]	Non-randomised experimental study	To assess how women with borderline personality disorder engage with their 12 to 18-month-old infants in separation– reunion episodes	Mother infant Dyads. Cohort 1: 12-month-old infants. 10 mothers with BPD; 22 control mothers without psychopathology (*n* = 32). Cohort 2: 27 mother-infant dyads, where the mothers had BPD	Modified set situation was applied to each mother infant dyad. Still face phase (90 s). Rapprochement and spatula phase then administered. Rating of whole situation then applied	Administration of validated measure
Hulette (2011), USA, Journal Article [60]	Cohort study	Investigate the intergenerational relationships between trauma and dissociation	67 mothers and their children aged 7–8 years old. 36 boys, 31 girls	Brief Betrayal Trauma Survey (BBTS) and Brief Betrayal Trauma Survey—Parent report (BBTS—Parent) administered. Categorised based on responses to 'high betrayal trauma', 'low betrayal trauma' or 'no betrayal trauma' conditions. Parents dissociation assessed via Dissociative Experiences Scale (DES). Children's dissociation was assessed by the administration of the Child Dissociative Checklist (CDC)	Administration of validated measure
Kiel (2011), USA, Journal Article [21]	Non-randomised experimental study	investigate the association between borderline personality pathology and at-risk parenting. Investigation the nature of parenting in response to infant distress in mothers with and without borderline personality pathology	99 infants and their mothers; divided into ''high borderline personality" and "low borderline personality groups". Infants range from 12–23 months	Mothers screened with Borderline Evaluation of Severity over Time (BEST); Difficulties in emotion regulation scale (DERS); Depression, Anxiety, Stress Scales (DASS-21); 1180 s of reunion in the "Strange situation" was coded. Infant affect, maternal affect, maternal behaviour were all scored. Demographic testing revealed no significant differences between high BP and low BP conditions. Micro-analysis of interactions	Administration of validated measure
Lewis (2020), USA, Journal Article [61]	Non-randomised experimental study	Examining parent factors related to changes in dissociation symptoms in childhood	68 Mothers (‘likely BPD’ and other) and their preschool aged children	Dyads completed assessment two years apart (T1 and T2). 2.5 h assessment session. Mothers completed a battery of self-report questionnaires about their and their children’s mental health symptoms. Mothers administered DERS at T1 and T2; DES administered at T2 only Child dissociation was assessed using a modified subscale for the Child Behaviour Checklist at T1 and T2	Theoretical discussion with explicit theoretical basis
Liotti (2004), Italy, Journal Article [36]	Non-systematic review and opinion	Review findings suggesting that disorganised attachment is central to trauma-related disorders, and dissociation is related to disorganisation of early attachment	N/A	Summary and synthesis of evidence and theoretical opinion	Theoretical discussion with explicit theoretical basis
Liotti (2009) Italy, Book Chapter [62]	Non-systematic review and opinion	Book Chapter. Discussion of the relationship between attachment and dissociation	N/A	Summary and synthesis of evidence and theoretical opinion	Theoretical discussion where there is no explicated model
Lyons-Ruth (2012), USA, Commentary Article [27]	Non-systematic review and opinion	Commentary article on Stepp, Whalen, Pilkonis, Hipwell, and Levine (2011)	N/A	Response article	Theoretical discussion
Macfie (2014), Spain, Book Chapter [63]	Non-systematic review and opinion	Review of evidence whether BPD has its origins in part due to failure to negotiate early childhood tasks, focusing on the role of parenting	N/A	Summary and synthesis of evidence and theoretical opinion; review of evidence for role of parenting in aetiology of BPD	Theoretical discussion with explicit theoretical basis
Mosquera (2014), Spain, Journal Article [64]	Non-systematic review and opinion	Explicate pathway(s) from attachment disruption to specific symptomatology in BPD patients	N/A	Summary and synthesis of evidence and theoretical opinion; integration of the Theory of Structural Dissociation of the Personality with mother-infant attachment in BPD context	Theoretical discussion with explicit theoretical basis
Mucci (2021), Italy, Journal Article [65]	Non-systematic review and opinion	To clarify the effects of intergenerational psychological traumatization, stratifying into two levels: 1) human agency (lack of attunement) and 2) actual abuse, maltreatment or incest as seen in Borderline pathology	N/A	Summary and synthesis of evidence and theoretical opinion	Administration of validated measure
Ozturk (2006), Turkey, Journal Article [66]	Cohort study	To assess the dissociative experiences, including borderline personality disorder, among first degree relatives of dissociative patients	24 Dissociative patients (18 diagnosed with DID; six with DDNOS). 50 family members, first degree relatives, of these patients were contacted	Family members administered Dissociative experiences scale Structured Clinical Interview for DSM-III-IV Personality Disorders (SCID-II) Borderline Personality Disorder Section—Turkish version Childhood Trauma Questionnaire (CTQ) Three (3) family members who had a DES score > 25 were administered the Structured Clinical Interview for DSM-IV Dissociative Disorders (SCID-D)	Administration of validated measure
Reinelt (2014), Germany, Journal Article [25]	Cohort study	To test longitudinally and in a community-based sample if maladaptive mother–child interactions (conceptualized by an insensitive parenting style and discrepancies in the perception of psychopathological problems of the offspring) mediate the relationship between maternal borderline symptomatology and BPD symptoms of the offspring about 5 years later. (p.11)	230 families comprising 295 Children and their biological mothers; all families involved in the Griefswald family study	Assessed with instruments at two timepoints; initial time T0 and T1 (approx. 5 years on). Maternal BPD symptoms assessed with the self-rating part of the German version of the SCID for DSM-III-R (SCID-II) Perceived insensitive habitual parenting style of the mothers, adolescents completed the EMBU ('own memories concerning upbringing') scale. Self-report questionnaire consisting of three scales (rejection, emotional warmth, and overprotection). 4-point Likert scale. Primary caregiver filled out the Child Behaviour Checklist (CBCL) and offspring filled out the corresponding Youth Self Report (YSR). BPD symptoms of adolescents / young adults were examined using the German version of the SCID-II interview for DSM-IV	Theoretical discussion with explicit theoretical basis
Schore (2001), USA, Journal Article [67]	Non-systematic review and opinion	To describe the negative impact of traumatic attachment on neurodevelopment and infant mental health, summarise the neurobiology of infant trauma and the neuropsychology of disorganised / disoriented attachment	N/A	Overview of the neurobiological consequences of early relational trauma on brain development, affect regulation and infant mental health including dissociative response and issues	Theoretical discussion where there is no explicated model
Stepp (2012), USA, Journal Article [68]	Non-systematic review and opinion	To describe the parenting strategies that might explain transmission of intergenerational trauma from mothers with BPD to their offspring	N/A	Summary and synthesis of evidence and theoretical opinion; parenting strategies (behavioural) and their relation to transmission of trauma from BPD mothers to their offspring	Theoretical discussion where there is no explicated model
Zalewski (2014), USA, Journal Article [26]	Cohort study	To examine the associations between maternal BPD and parenting of 14-17yo girls, and their mothers through assessment of several different cohorts longitudinally	Girls from the Pittsburgh Girls Study (PGS, *N* = 2,451). Urban community, girls aged 15–17; biological mothers and their adolescent daughters *n* = 1,598. 3 cohorts, collected in 3 waves from 7, 8, 9 years – 15,16, 17 years. Biological mothers only sampled	Tests administered at 1-year intervals. In home interviews conducted separately by trained interviewers. Mothers reported on own psychopathology and their daughters' temperaments. Daughters reports on their parents' use of psychological and behavioural control. Parent Behaviour Inventory. Three subscales were intrusiveness, control through guilt and acceptance of individuation. Behavioural control: parent report on the Conflict Tactics Scale: Parent–Child Version Maternal BPD symptoms: The International Personality Disorders Examination Negative emotionality: parent report when girls were age 15; used emotionality, activity, and sociability temperament survey	Theoretical discussion where there is no explicated model

Descriptive analysis of the data was also undertaken to better reify results in respect to research questions. Through this process, data was organised additionally in respect to measures of dissociation, where a measure was articulated (Table 2), and in reference to each study’s articulation (or lack thereof) of a theory of dissociation, and the impact of this articulation on the study and results interpretation (Table 3). The creation of these tables was based on similar scoping reviews answering theoretical questions [69]. Finally, given the aims of the study to map the evidence regarding dissociation and its interest in the literature as a variable of concern, both for intervention and as a possible mechanism for intergenerational transmission of trauma, a final table was created to synthesise results in respect to these questions where the study offered a definition or theory of dissociation (Table 4). No risk of bias assessment was conducted. This was in accord with [51] guidelines regarding scoping reviews and is further discussed as a limitation of the current study.

**Table 2:** *Measures used to assess dissociation in adults (n* = *11)*

Measure	First Author, Date	Type	*N*	Description of Measure
Dissociative Experiences Scale (DES)	Hulette, 2011; Lewis, 2020; Ozturk, 2006	Self-Report	3	Self-report measure comprising 28 items measuring continuum of dissociative experiences
Atypical Maternal Behavior Instrument for Assessment and Classification (AMBIANCE)	Haltigan, 2019; Hobson, 2009	Standardised Observational	2	Detailed observational coding protocol comprising 150 items over 5 dimensions of disrupted maternal behaviour
FR coding system*	Hesse, 2000	Self-developed Observational	1	Observational coding system; not clearly reported (from doctoral dissertation)
Micro-assessment, no standardised measure	Crandell, 2003; Hobson, 2005; Kiel, 2011; Macfie, 2014	Self-developed Observational	4	Various protocols including moment-by-moment observation and coding
Structured Clinical Interview for DSM-III-R (SCID-II)	Reinelt, 2014	Structured Clinical Interview	1	Structured Clinical Interview standardised to DSM-III-R for assessing presence of BPD symptoms, including Dissociation

**Table 3:** Theories of Dissociation identified

Theory of Dissociation	First Author, Date	N	Description of Theory
Attachment-based	(Liotti, 2004; Liotti, 2009)	2	Proposes dissociation is in itself a painful experience that occurs interpersonally rather than intrapsychically and disrupts caregiving
Relational Psychoanalysis	Blizard, 2003	1	Proposes dissociation as a mechanism for disrupted caregiving (mother) and to deal with ‘double-bind’ situations whereby the child is subject to seeing its caregiver as hostile or aggressive
Theory of Structural Dissociation of the Personality (TSDP)	Mosquera, 2014	1	Proposes that dissociation happens intra-psychically and splinters the personality in such a way that it become impossible to access certain ‘parts’ of the personality, usually divided into ‘emotional’ and ‘apparently normal’ parts, leading to disrupted caregiving in those who have dissociated ‘parts’ of the personality
Diagnostic and Statistical Manual (DSM)	Hulette, 2011*; Lewis, 2020; Ozturk, 2006	3	Dissociation is defined broadly as any interruption to *subjective* integration of various mental systems (behavioural, emotional, sensory etc.), which may lead to lapses in effective parenting
Neuropsychoanalysis	Mucci, 2021	1	Dissociation is located as a right-brain-hemispheric phenomena that occurs in respect to ‘relational’ trauma of a pre-oedipal kind (i.e. developmentally early). Dissociation is seen as an intrapsychic defence against overwhelming emotions that are triggered in infants in response to human-inflicted trauma. Dissociation then operates unconsciously and pre-verbally and is associated with internalisation of split-off ‘victim-persecutor’ internal working models, which then govern emotional response to others including in a caregiving setting i.e. in BPD adults providing care for offspring
Betrayal Trauma Theory*	Hulette, 2011*	1*	Dissociation most likely to occur in those who have a close relationship to the perpetrator. In mothers who have BPD or suffer high betrayal trauma and dissociation, the theory states that the awareness of external threats to their offspring may be diminished due to overreliance on defensive dissociation to deal with stressful or affectively salient stimuli and situations
Psychoneurobiology	Schore, 2001	1	Dissociation is discussed in terms of a neurobiological defence against metabolic dysregulation occurring as a part of stress cascade responses in the face of unregulated emotional activation. The neurobiology of dissociation in infants is discussed, as well as the interpersonal consequences of dissociation and the long-term effects of (infant) dissociation. Maternal dissociation is an automatic blunting response, primed from infancy, and disrupts infant attachment through ‘suboptimal neurobiological priming’ i.e. Mothers are unable to effectively regulate their own offspring due to their own dissociative responses
No theory offered but dissociation subsumed under other issues	Hesse, 2000; Reinelt, 2014	2	Dissociation is seen as a symptom of BPD but not necessarily discussed as a causative agent of mother–child disruption
None (not explicated)	Crandell, 2003; Haltigan, 2019; Hobson, 2005; Hobson, 2009; Kiel, 2011; Lyons-Ruth, 2012; Macfie, 2014; Stepp, 2012; Zalewski, 2014	9	Dissociation is implied or explicitly addressed, but no definition is offered for the construct by the authors

^*^This record is included twice as the authors use a DSM definition of dissociation, but also reference their own theory (betrayal trauma) to account for the development of dissociation. Further discussion is provided in the results section below

**Table 4:** Theoretical understandings of dissociation and future directions

	**Blizard 2003**	**Hesse 2000**	**Hulette 2011**	**Lewis 2020**	**Liotti 2004**	**Liotti 2009**	**Mosquera 2014**	**Mucci 2021**	**Ozturk 2006**		**Reinelt 2014**	**Schore 2001**
Was the understanding of dissociation coherently and adequately described?	Yes	Yes	Yes	Yes	Yes	Yes	Yes	Yes	Yes	Yes		Yes
Was dissociation a target for measurement?	N/A	Yes	Yes	Yes	N/A	N/A	N/A	N/A	Yes	Yes		N/A
Was dissociation described as a target for future study?	No	Yes	Yes	No	Yes	Yes	No	No	No	Yes		No
Did authors describe how their results/theory relate to dissociation?	Yes	Yes	No	No	Yes	Yes	Yes	Yes	Yes	Yes		Yes

### Thematic analysis

A thematic analysis was conducted to evaluate qualitative data, particularly in relation to the role of dissociation in intergenerational transmission of psychological trauma. Given the broad research questions, whole studies were analysed to generate an understanding of how researchers conceptualised, tested, and thought about dissociation and its relevance to intergenerational trauma in the BPD population. The analysis followed an essentialist / realist paradigm but focussed on both the semantic and the implicit level of data; meaning within the text was garnered from both the reported data, and examined at an implicit level in reference to variables of concern. For example, in studies where observation of caregivers was conducted such as Kiel et al. [21], a lens of ‘dissociative’ caregiving was applied, and content examined for latent discussion of the concept(s) of interest.

The position adopted in this paper acknowledges that different perspectives may be used to construct an understanding of underlying phenomena. The reporting in each included source was thought to be subject to contextual factors such as: researcher’s theoretical stance in relation to the observed construct(s), methodology of each study, including measurement and assessment of dissociation, and broader contexts such as academic climate and purpose of the research. The current study therefore sought to advance knowledge of dissociation by contrast of sources with the aim to produce further basis for testing and refining of knowledge of the objects in question; dissociation in the BPD caregiver context. Although outside the scope of this paper, this approach is compatible with offering meta-theoretical perspectives on the constructs of interest and is predicated on the idea that there is an essential nature of dissociation, and a’real’ impact on caregivers and their interactions with children.

The thematic analysis followed [70] and was conducted in the above epistemological paradigm. Within this frame, the researchers looked at latent and manifest themes using a theoretical thematic analysis; a non-traditional but not mutually exclusive set of paradigms ([70], p. 14). For example, each study was analysed into broad themes; results were then compared to each other study by study, and studies were reviewed with updated information from other studies. In this way, studies that cursorily mentioned dissociation, but met inclusion criteria through manifest content reflective of latent ‘dissociation’ were given as much attention as where dissociation was a variable or theoretical construct of explicit concern. Updated information was utilised dynamically to re-review material, creating an iterative approach to the dataset, and therefore allowing a more comprehensive analysis of the literature, i.e. if one study offered a perspective of dissociation as a defence to psychological pain, other results were re-reviewed looking for suggestion that dissociation was used in the same context, or not. This approach was chosen due to the heterogeneity of the construct(s) and the purpose of the scoping review, and it was thought to allow the ideas to be mapped in a coherent fashion.

Utilising the above set of principles, the data (the included texts) were analysed in phases consistent with Braun & Clarke’s [70] paradigm: 1) the data was read and re-read; 2) all relevant passages were extracted and formed the raw ‘data’ for the analysis; 3) those data were contextualised and coded (labels generated) in respect to their categorisation of dissociation (including function, aetiology and impact, as well as the way in which dissociation was talked about); 4) the data was re-searched for the coded ‘themes’ in order to cross reference and iteratively generate a consistent ‘map’ across studies of relevant themes. New themes were identified when coded data were repeated across studies and where coded material both offered a different perspective to information already identified (‘themed’) whilst simultaneously constituting a discrete entity within the data. For example, coded data that contrasted (e.g. dissociation being defined under conflicting paradigms) was subsumed under a theme of ‘dissociation as confusing’; separate themes were only identified when the data was unrelated rather than contrasting or conflictual. Researcher bias was considered in that the researcher’s perspectives and readings actively constructed the themes represented. This was seen as a strength and limitation of the study (explicated below in the discussion). Themes generated in the analysis were therefore influenced by the lead author’s synthesis of disparate schools of thought, as well as recognition of fundamental differences between meta-psychological paradigms.

## Results

### Study selection

Of the 5,087 titles and abstracts examined, 236 full-text papers were screened, with a final 20 records meeting the criteria for inclusion in the review (see Fig. 1). Due to conceptual lack of clarity around BPD and dissociation, some records with differing measures and definitions were included as their definitions of constructs under investigation were consistent with the constructs of interest to the present study. Additionally, all records using the AMBIANCE measure were manually reviewed (*n* = 46). The AMBIANCE is an observational measure of parent–child interaction designed to assess behaviours in at-risk mother–child dyads. One of the five domains of maternal behaviour assessed is the ‘fearful/disoriented’ subscale. This subscale represents a division of the theoretical construct proposed by the work of Hesse and Main [39], where they group ‘frightened / frightening’ behaviours, and argue that these behaviours contribute to infant disorganisation when the mother displays them to the infant. The ‘fearful/disoriented’ subscale subsumes the ‘frightening’ behaviours from Main and Hesse, which are thought to contribute to infant disorganisation. These behaviours are possibly dissociative behaviours and thought to represent dissociative intrapsychic processes in the mother, thus, these records were reviewed as they were conceptualised to fundamentally assess dissociative behaviour in caregivers, whether or not the definition of the construct under investigation in the study was explicitly labelled as dissociation. A summary of measures and study designs are outlined in Table 1. All studies were published in peer-reviewed journals, other than two which were published book chapters [62, 71]. There were three studies [56, 58] that used the same study participants and data, in addition [59] used this data and a second cohort.

### Study characteristics

Of the 20 studies included, 10 (50%) of the records were completed in the United States, 3 (15%) in Italy, 3 (15%) in the UK, and 1 (5%) each in Canada, Spain, Turkey, and Germany. Three studies (15%) were published in the last 5 years, with the oldest study being published in 2000. Ten studies (50%) were experimental. Of these experimental studies, 6 (60%) were mixed methods quasi-experimental designs, 3 (30%) were mixed methods cohort studies, and 1 (10%) was a latent-class analysis of a measure. Of the 10 experimental or quasi-experimental studies, all of them included administration of measures to mothers and their offspring at different timepoints; five (50%) were conducted with mother-infant dyads, three (30%) with mother–child dyads, and one (10%) with mother-adolescent dyads and one (10%) with unclear ‘family-child’ dyads where it was not reported who was the relevant caregiver (mother, father, or other). Sample sizes in the quantitative studies ranged from 32 to 1,598. Of the 20 included studies, only eight (40%) offered a definition of dissociation and theoretical model.

### Theoretical understanding of dissociation in the target population

In line with the methods outlined above the theoretical understandings of dissociation in caregivers with BPD was mapped (see Table 3 for details on dissociation theories). Nine of the 20 included sources (45%) offered no theory of dissociation or definition of the construct. Of the remaining 11 sources, Table 3 shows the breakdown of theories. The most common definition or theory of dissociation offered was a DSM definition of dissociation (*n* = 3, 28%). The first study in this category [60], investigated the role of dissociation in a population of mothers and their children. The study was included despite its lack of overt reference to BPD, due to its presence in the broader literature as positing a theory of concern for the effects of longitudinal transmission of trauma within the parents and children [72, 73]. This study was categorised as having a DSM definition of dissociation; however, it was apparent dissociation could be subordinate to ‘Betrayal Trauma Theory’ in this source. For this reason, the record was included twice in Table 3. The second study, [61], measured and investigated dissociation from a DSM definition basis, but focussed on maternal ‘dissociative behaviour’ as measured by the Dissociative Experiences Scale (DES; [74]), a self-report scale related to dissociative experiences. There were several methodological issues with this study, identified by the authors, and discussed further in the discussion section. The final source [66], investigated dissociation as the variable of concern, again using the DES but in a broader family context. The study met inclusion criteria as it investigated dissociation as a variable of concern and offered hypotheses on different ‘types’ of dissociative family structures that may contribute to psychopathology in offspring. Dissociation, although contextualised in theory with the DSM definition, appeared to take on a different ‘meanings’ throughout the source; this is more fully explored in the discussion section below. For full information on measurement, theoretical perspectives, and aims of included sources, see Tables 1, 2 and 3.

The next most common theoretical position was to subsume the topic under other constructs (*n* = 2, 18%). Of these two sources, dissociation was subsumed in one [25] as a symptom of BPD with no further discussion or definition. The BPD diagnosis was concordant with a DSM characterisation; however, the source was found not to provide a theoretical position as there was no definition of dissociation offered. Thus, dissociation was a symptom of BPD, and no theory was provided to understand how or why dissociation occurs. The second source, [39], used dissociation to conceptualise a pathway of intergenerational transmission of trauma, and subsumed dissociation under their own development of a coding criteria to classify parental dissociative behaviours, along with frightening or frightened behaviour (referred to as ‘FR coding’). This was a novel argument regarding the theoretical and practical importance of measuring dissociative behaviour however no formal definition was offered; hence this was deemed to be ‘subsumed’ into their own construct (FR behaviour), which has since become clinically important in the literature.

Following this, two sources (18%) by the same author [36, 62] utilised Attachment Theory to locate dissociation, focussing on the nature of dissociation in relation to attachment relationships. The authors advance a theory of dissociation that places attachment relationships as central to a certain type of dissociation. Both sources also presented a psychoneurobiological understanding of dissociation, however attachment theory was used to conceptualise the importance of dissociation, and the psychoneurobiological correlates or underpinnings were offered as evidence of the causes and effects of dissociation within this context; hence the sources were viewed as locating dissociation primarily in an attachment context.

One source (9%) identified dissociation as a neurobiological event and provided robust description and discussion of the mechanism. Schore [67] offered a basis for understanding dissociation as a mechanism and symptom in a paper concerned with theoretical advancement and integration of existing literature. Although the study did not focus explicitly on BPD, BPD (or a subset of BPD diagnoses) is conceptualised within the study as an adaptation to early adversity and seen as a dissociative process. The description offered of dissociation within caregiving populations is rooted in a broader understanding of ‘attachment theory’. However, given the interdisciplinary nature of the source and the thorough explanation of neurobiological mechanism and effects of dissociation, in relation to caregiving relationships, the neurobiological approach was seen as the dominant paradigm.

One source (9%) contextualised dissociation within a neuropsychoanalytic theoretical paradigm [65]. Mucci offers a perspective on dissociation as positing the importance of the mechanism for the development of borderline psychopathology and conceptualising the phenomena within a psychoanalytic framework integrated with modern neuroscientific research; neuropsychoanalysis.

One further source (9%) offered a specific framework, the Theory of Structural Dissociation of the Personality (TSDP) to organise thinking about dissociation and the role of dissociation in the target population [64]. In their theoretical position paper, they attempt a non-systematic review of evidence regarding BPD, attachment styles, emotion dysregulation and early life experience, integrating these ideas through the use of the TSDP.

Finally, one source (9%) used the perspective of relational psychoanalysis to provide a non-systematic overview, and advice on treatment, of BPD. They posit that dissociation is a key concept in the intergenerational transmission of trauma [55]. The author presents a summary of existing research and describes ‘double-bind’ situations where a child is confronted with threatening behaviour from an attachment figure, placing that child in a ‘double-bind’ of having no ‘safe’ route for meeting their needs. They hypothesize that this provokes dissociation in children, and that caregivers exhibit dissociated internal systems which contribute to inconsistent behaviour toward their offspring.

Of the remaining 9 sources that did not offer a theoretical basis for dissociation, and only casually or implicitly referred to the mechanism, four (45%) were non-randomised experimental (cohort) studies [21, 56, 58, 59]; two (22%) were non-systematic review and summary sources [63, 68]; one (11%) was a latent-trait modelling analysis of an existing measure, the AMBIANCE [57]; one (11%) a commentary article on an included study ( [27]; commenting on [68]); and one (11%) was a cohort study [26].

Of the 20 included sources, none concerned intervention on reducing dissociation in caregivers, or the impact of intervention on child outcomes of attachment disorganisation or dissociation. Table 1 reports on findings related to dissociation from all 20 sources.

### Thematic analysis

To examine the relationship between dissociation in caregivers with BPD and the intergenerational effects on their offspring, as well as to understand how dissociation was understood theoretically by researchers, a thematic analysis was conducted. Of importance was the use, or disuse, of dissociation as an explicit and theoretically important construct, as well as observed, component of caregiver behaviour, or conversely as an implicit mechanism or driver of these processes. The presence of dissociation as an *idea* of concern in the broader literature, but relative lack of explicit focus, was particularly of importance; as well as the importance or lack thereof of dissociation for explaining other phenomena. Through the analysis, four distinct themes were developed. The four themes reflected the pervasive lack of caregiver perspectives across the literature; the importance of confusion as a core aspect of dissociative experience in the population and in caregiver – infant interactions; that dissociation was seen as a key aspect of intergenerational transmission of trauma in the population; and finally that dissociation was viewed as fundamentally being an interruptive process whereby a range of processes (development, language, relationships, perception of self and others) can be impaired by dissociation, both in caregivers and transmission to infants.

### Theme: lack of caregiver perspectives

The most frequently occurring theme was the focus displayed on description of infant experience and possible developmental pathways, rather than subjective or lived experience of caregivers. Most sources included dissociation as a caregiver variable of concern in reported results or theoretical discussion (*n* = 11), however there were notable difficulties identifying a clear theoretical and empirical underpinning of dissociative caregiver behaviour. Only 5 of the sources measuring dissociation directly offered a clear understanding of the construct.

The lack of clarity was observed in links being made in a variety of areas, but difficulty with further elaboration. Hesse and Main [39] suggest that dissociative caregiver behaviour leads to infant disorganisation. Hobson et al. [58, 59] propose and observe infant disorganisation as possibly suggesting dissociated states of mind. Liotti [36, 62] suggested that infant disorganised behaviours were similar (phenotypically) to adult dissociation. Crandell et al. [56] showed infants of BPD mothers displayed more ‘dazed’ looks, indicative of disorganisation of emotional processes, than those of healthy controls. Liotti suggests BPD caregivers are subject to developmental disorganisation, and that dissociative behaviour manifest in this group overlaps with the behaviour exhibited by disorganised infants. Lewis et al. [61] suggests an association between dissociation in mothers and harsher parenting practices, which are linked to development of disorganised attachment.

Across the sources identified, dissociation and disorganised attachment were clearly linked. Developmental lines were drawn regarding BPD and dissociation and Mosquera et al. [64] suggest BPD, or a subtype of BPD, may be a dissociative disorder. Despite all of these links between disorganisation, dissociation, and BPD, the lack of clear theory appeared to restrict focus, resulting in no elaboration of caregivers’ subjective experience. There was also a lack of information regarding whether dissociation was unique or differentiated in the attachment context as opposed to outside of it.

Infants experience was seen as painful to the point of employing ‘defence’ however the ‘pain’ of the caregivers was not explored. Lyons-Ruth [27] wonder if different parenting styles may be characteristic of different disorders and recommend a future course of study in this area, which suggests the importance of understanding the parent’s experience:

The availability of [parenting observational measures] opens an array of theoretical issues for further exploration, such as whether there are specific kinds of disruption in early communication (i.e. frightening vs. dissociative vs. role-confused) that are particularly characteristic of parents with different kinds of disorders. (p. 2)

However, the focus remains, at least in part, on the intersubjective quality of the parent in concert with the infant. The parent’s experience is marked as ‘dissociative’, but the subjective experience is lacking. Schore and Mucci [65, 67] refer to dissociation, both in caregivers and in offspring, as a ‘deadening’ process. Schore [67] suggests “Clinically, dissociation is described as “a submission and resignation to the inevitability of overwhelming, even psychically deadening danger” (Davies & Frawley, 1994, p. 65).” (p. 232).

Relatedly, Mucci [65] refers to dissociation as “affect deadening”, suggesting that attachment-based trauma causes both “…an impaired capacity to regulate stressful affect and an overreliance on the affect deadening defence of pathological dissociation” (p. 101).

The subjective quality of the mother’s experience is not investigated, nor her emotional state.

Liotti [36] similarly describes parents “with a ‘dead’ stare, unblinking, in the face of the infant’s cry for help” (p.478). Haltigan [57] include as an operationalisation of dissociative behaviour in mothers “Deadened or flattened affect leaving empty feel to interaction” (p. 263). Schore [67] quotes a chilling account of a mother and her baby,

During a testing session, her baby begins to cry. It is a hoarse, eerie cry . . . On tape, we see the baby in the mother’s arms screaming hopelessly; she does not turn to her mother for comfort. The mother looks distant, self-absorbed. She makes an absent gesture to comfort the baby, then gives up. She looks away…In the background we hear Mrs. Adelson’s voice, gently encouraging the mother. “What do you do to comfort Mary when she cries like this?” (The mother) murmurs something inaudible. . . As we watched this tape later . . . we said to each other incredulously, “It’s as if this mother doesn’t hear her baby’s cries.” (cited in Barach, 1991, p. 119) (p.218)

Although illustratively helpful, the mothers’ perspective is lacking. It is as if she is not considered – or considered only as an extension of her baby. Indeed, none of the included sources investigate the subjective experience of caregivers, other than providing a descriptor of absence. The lack of subjective description may be linked to assumed difficulties with memory integration when dissociated, however without an understanding of any subjective experience, the pain of the caregivers becomes relegated to non-existence also.

### Theme: confusion as a core component of dissociation

Researchers appeared to classify dissociation in a variety of ways, promulgating confusion and demonstrating the confused nature of the subject material. Some authors referred to dissociation as occurring on a continuum and phenomenologically heterogenous; for instance, Mucci [65] addresses this directly.

Dissociation is certainly neither ‘‘deliberate and intentional,’’ nor closer to the conscious spectrum and, in fact, manifests itself in a continuum of severity up to the level of actual confusion between reality and unreality, as in psychosis. (p.88)

Mucci suggests that dissociation occurs on a spectrum and underpins other diagnostic considerations. A different understanding is offered by Liotti [62].

These longitudinal studies provide strong support for the contention that pathological dissociation should not be viewed as the top end of a continuum of dissociative experiences ranging from normality to psychopathology, “but as a separate taxon that represents an extreme deviation from normal development” (Ogawa et al., 1997, p. 855). (p.58)

The issue of taxonomy of dissociation is addressed in part by these two authors; however, in many of the sources, there was no explicit attempt to explore. Where there was no explicit discussion, multiple instances of conflicting use were found within sources. This confusion in respect to taxonomy was found across sources, where assessment, measurement and classification of dissociation was avoided. The confusion in classification was reflected in the core nature of dissociation as being an inherently confusing experience. Macfie et al. [63] suggest confusion is a core component of dissociative experience in offspring of caregivers with BPD,

…and in the domain of self-regulation they display more narrative incoherence, confusion between self and reality, confusion between self and fantasy, and fantasy proneness, the latter three being associated with dissociation (Macfie & Swan, 2009). (p. 19)

And they make the explicit link to this, unresolved narrative representations, and the development of BPD, “These representations may be transmitted from one generation to the next with implications for the development of BPD” (p.19). Dissociative experiences are seen as confusing, and this confusion makes classification difficult. Dissociative behaviours may necessarily create a logical injunct due to enactment of conflicting behaviour. That is, insensitive behaviour may be dissociative on the part of the caregiver, and trigger dissociation on the part of the child, as the response offered by the mother is not contingent to the child’s need. This confusion was therefore identified as a core component both of dissociative experience and appears to make the identification and classification of dissociation inherently difficult.

### Theme: role of dissociation in intergenerational trauma

Authors discussed, implicitly and explicitly, dissociation at a variety of levels, all impacting trauma responses. There were suggestions that various ‘things’ could be dissociated within, or between, different ‘systems’. Dissociation was generally seen to act like a switch in a network of nodes, where each node had smaller ‘sub-nodes’, which each again could be subject to dissociative forces. At the most elementary level dissociation was described as a neurobiological process. This focus was adopted by two authors [65, 67]. Schore [61] addresses a section titled “The neurobiology of the dissociative defence”, putting in stark relief the import of locating and defining dissociative process. Mucci [65] also provided a neurobiological basis, but then frames dissociation in pluralist terms:

When the hyper-aroused state continues the child might detach from the world through dissociative responses (depersonalization, derealization, numbing, total passivity, and restricted affect). (p.99)

Mosquera [64] provided some location of dissociative processes as being neurobiologically based but does not offer further review.

At another level of abstraction, Otzurk [66] suggest dissociation occurs within a family unit, in a “social function” (p. 294). Further “In addition this asymmetry in dissociative psychopathology among family members may be seen as a system which itself is dissociated.” (p.294). The authors further define eight ‘family types’ that may appear on the surface ‘Apparently Normal’. Dissociation is seen as a lens that frames all these types of family.

“Dissociative family. Any one of the family types defined above may cover the characteristics of a dissociative family. It is common in this type of family to have at least one family member with a dissociative disorder or subclinical dissociative experiences... There are polarized roles in the family and a reversible abuser-victim cycle is common” (p.299)

Dissociation is used to define the pathology of a family member, the process occurring in the family leading to dysfunction, and families as a whole.

Another level of dissociation was found at the socio-cultural level. Dissociation was described in reference to historical import [65], and also as a cross-culturally stable construct [66], whilst simultaneously being suggested as being replaced as the dominant mechanism enabling traumatic responses by ‘repression’ due to cultural forces (e.g., Freud and his publication: *The interpretation of dreams*).

Dissociation was therefore seen as being studied at, and framed within, different levels or systems. Behavioural systems, attachment systems, neurobiological systems, family systems and socio-cultural systems were all seen as ‘dissociable’, all of which were thought to impact the individual’s ability to relate and their psychopathology in general. Researchers discussed dissociation in all of these realms, and implicated dissociation in playing a pathogenic or pathologic role within each of these systems.

### Theme: dissociation as a process of interruption

Authors discussed dissociation in relation to the interrupting nature of the phenomena. Dissociation in caregivers with BPD was seen to disrupt internal processes; [55] “Lyons-Ruth (1999, 2001a) proposed that dissociation may result from disconnections between procedural, enactive, “how-to” knowledge, and narrative knowledge, as well as among various systems of enactive knowledge.” (p. 36).

There is a discussion of dissociation as being a result of fundamental (internal) disconnections; memory is seen as disconnected. Dissociation is predicated on a process of interruption of connection in an intrapsychic sense. Similarly, dissociation was related to spatial and temporal dynamics, applied, and described in order to elucidate disruptions occurring in, or between, caregiver-child dyads.

Other researchers discussed the interruptive nature of dissociation in regard to language systems. In reference to interviews conducted with mothers about their experiences of childhood, [39] suggest:

We have proposed that such conversational/linguistic slips may be attributable to unintegrated or partially dissociated fear aroused by the discussion of these interview topics, and that anomalous forms of threatening, dissociative, and fearful behaviour may occur at times in (otherwise “normal”) parents. (p. 1102)

Notable in their description is the use of dissociation in relation to intra-subjective situations “partially dissociated fear” (p. 1102), and in the object “The parent might exhibit anomalous forms of threatening, frightened, or overtly dissociated behavior” (p. 1114). Dissociation is seen as a process that is observable in language of the subject due to disruption caused by dissociative actions of the object. [61] directly addresses the consequence in language of dissociation for the subject.

While dissociation may serve this protective function for children in at-risk environments, over time dissociative behaviors often have a negative impact on functioning, as demonstrated by its link with the onset of mental health problems and impaired functioning in early cognitive and language processes (Eisen & Lynn, 2001; Panzer & Viljoen, 2004). (p. 204)

The focalisation of dissociation as a process of interruption internally; of memory, language and externally; relationships (between subject and object), as well as disrupting development (temporality), and spatiality, creating ‘split-off’ parts of the self or ‘layers’ was consistently referred to throughout the included texts. Some authors [55, 65, 67] when suggesting treatments or therapies addressing dissociation suggested that the interruption caused by dissociation need to be directly addressed in order to provide therapeutic benefit, and that psychotherapeutically the therapist would “…serve as a relational bridge between dissociated self-states” ( [55], p. 28), furthering the use of spatial metaphors to describe the tendency of dissociative processes to cause interruption between dyads, and the need to address dissociation through human relationships.

## Discussion

This scoping review aimed to summarise sources regarding the intersection of BPD, Dissociation, and parenting capacities; particularly understanding the research base regarding parents with BPD who dissociate, and the effects of dissociation on offspring. Key questions regarding how researchers conceptualised parental dissociation, the role of dissociation as a mechanism for transmission of intergenerational trauma within the BPD context, and the state of intervention studies in the area, were all addressed through analysis of sources and thematic analysis.

When considering the way in which dissociation was discussed, the predominant finding was the lack of a unified theoretical approach. The sources ranged in their theoretical integration between having no defined theory of dissociation and the mechanism being implied, to full accounts of the neurobiological underpinnings of the mechanism. Those who did offer a description of dissociation differed in categorisation of dissociation. It was unclear whether dissociation in the BPD caregiving context represented a separate taxonomy (as distinct from ‘everyday’ dissociation, or dissociation occurring in other psychopathologies or contexts), or whether dissociation occurs on a spectrum but fundamentally arises from the same (neurobiological) mechanism. Given this, the measurement of dissociation in this population appears to be a significant issue that has received relatively little empirical attention. It was unclear which measures assessed specific taxon’s of dissociation. Thus, a specific attachment based taxon could be defined/referred to as “relational trauma”, existing as an intersubjective interpersonal context. Such a definition could lead to exploration of ways of measuring dissociation in this context, where activation of relevant neurobiological systems is necessary in order to ‘provoke’ the dissociation. Therefore, measures such as the DES, frequent in sources identified, may be ineffective at sensitively registering the dissociation that might affect BPD parent–child dyads. Observational measures where attachment behaviours are elicited or expected may therefore reflect a more clinically relevant taxon of dissociation. Other novel ways of eliciting this activation and understanding of dissociation should be sought (such as those offered by [40, 75]) and compared with measures in a non-attachment context in order to understand this relationship and effectively screen mothers with BPD for their propensity to dissociate. Such understandings may have implications both to the risk of attachment disruption of their infant and to the efficacy of psychotherapeutic interventions to address the health of the dyad.

Next, we found that dissociation presented conceptual difficulty for researchers. This meant that dissociation was operationalised differently between sources, leading to the construct being subsumed under other constructs. The general body of sources identified theoretically that dissociation is a) likely present in BPD caregivers, b) BPD caregivers are more likely than other populations to have children who have disorganised attachment, and c) children with disorganised attachment are likely to experience dissociation when they are older. The empirical work needed to test association and causation within the BPD context was sparse. The logical inferences suggest that offspring of BPD will be more likely to experience dissociation. However, it is unclear why, or what impact this has on their caregiving ability as distinct from other populations. For instance, the included study by [25] found, through path analysis, that dissociation mediates the relationship between maternal BPD symptoms and offspring BPD symptoms through parenting behaviours; however not as strongly as ‘internalising symptoms’ in mothers. Internalising symptoms were conceptualised in the same study to exclude ‘dissociation’, as dissociation (measured by the DES) was seen as a separate construct, a symptom of BPD. Internalising symptoms however may have a basis in dissociative mechanisms, leading to conceptual confusion. Re-conceptualised, and integrating the various theories identified in the sources of this review, it was plausible that dissociation is represented in all ‘internalising’ behaviours, particularly difficulty identifying feelings, which was found to be a significant path mediator in the same [25] study. This lack of an integrated model was highlighted across the sources.

The importance of dissociation as underpinning other BPD symptoms is made more likely by the identification of the disruptive effect of dissociation on language, and that dissociation is represented through language; another theme that was generated in the current study. Researchers spoke about dissociation as having a disruptive effect, and noted particularly across sources that language disorganisation was a key sign of dissociation. This is concordant with broader research regarding the disruptive effects of dissociation neurologically on language processing [76–78]. The theme of disrupted language in the population suggests recursive links to other themes regarding methodology in studying dissociation in a BPD context. That is, if language is disrupted through dissociation (of a certain taxon), then the ability for caregivers to self-report or identify their difficulties may be disrupted also. The importance of identification of dissociative type BPD presentations in this context becomes more important, as self-report may become unreliable. Some researchers [37] suggest BPD is a dissociative disorder, where others [38] suggest a subtype of BPD where dissociation is the pathogenic agent and main feature. In the current study, dissociation was seen as expressed and observable through language of caregivers, however subjective self-report and observable behaviour were often not experimentally compared, suggesting a direction for further investigation. Future studies directed at testing these connections may help to further articulate specific behaviours, emotions or intersubjective phenomena that might lead to such disruption in the attachment relationship, helping to develop specific intervention for this vulnerable population.

The review also attempted to investigate interventions on dissociation in caregivers with BPD. Of the included sources, seven (35%) made reference to intervention [27, 36, 55, 62, 64, 65, 67], None of these studies offered empirical accounts of intervention, however theoretical discussions were provided. The sources focussed on pathogenesis of dissociative experiences through relational trauma, and treatment directions were oriented towards adults with dissociative experiences rather than caregivers with dissociative experiences per se. Across the review, there were no studies that offered a unique perspective on treatment of dissociation in caregivers specifically, although theories provided could be useful to help the target population. All studies however highlighted the importance of the therapeutic relationship as being important in addressing dissociation. Taken together, a significant gap and direction for future research may be investigating direct effects of dissociation on therapeutic effectiveness in the target population. Given the importance of the therapeutic relationship in addressing dissociation, a potential avenue for intervention may be to leverage the attachment relationship developing between the caregiver and child in order to address dissociation. There were however no studies that attempted mother–child dyadic therapy in the sources reviewed.

Finally, given the lack of clarity in the area identified in the current review, the researchers would like to offer a provisional operationalisation of dissociation in the target population. Many articles offered a position (although in different theoretical languages) of dissociation being employed as a response to overwhelming affect aroused in the attachment context. This ‘defence’ is initially employed by the infant but later with children and adolescents, specifically to deal with a hostile or frightening caregiver. In the case of older children (and adults), the defence can be employed to deal with subsequent ‘internalisation’ of such a figure. Thus, the link between initial ‘disorganisation’ in attachment relationship(s), and the subsequent development of dissociative behaviours, evident in BPD, is established.

The researchers would endorse the perspective that in the BPD caregiver context dissociation be defined as a re-activation, in the ‘revived’ attachment context (now as a parent), of an unconscious neurobiological process. All dissociation in this context would be thought to be reflective of ‘trauma’. The trauma referred to is the nature of overwhelming experience; dissociation can be conceptualised to only occur when the environment provides failure great enough to threaten ‘overwhelming’ of other capacities to remain in a metabolically stable state. When there is a failure of ability to self-regulate, and a failure of coregulation, dissociation remains an available option. This process is later employed to protect against interpersonal experience and intrapsychic experience of ‘unthinkable’ content related to originally experienced, non-contingent, and possible hostile caregiving. The degree of reliance on the caregiver (i.e. if there are other available figures and if the caregiver is the primary attachment figure) would be thought to predict at least partially the original severity and frequency of dissociative response.

In the BPD caregiver it is possible that the closer or more ‘`like-me’ the infant and their needs, the greater the threat of reactivation of qualia of painful experience and therefore the more likely dissociation will be employed. Dissociation is primarily a neurobiological response and may manifest in multiple behaviours; all of which would be presumed to inhibit activation of painful attachment experiences. In this way, dissociation may exhibit in this population in a variety of caregiving behaviours, even ones that are classically ‘adaptive’ or helpful; the important factor would be presumed to be the non-contingency of the behaviour to the infants’ needs. Ultimately however, what is unique to dissociation in a BPD caregiver context will require further investigation with appropriate definition and measures of dissociative symptoms as well as interactional quality between caregivers and their children. Studies addressing these factors in concert may hope to further clarify the nature and effects of dissociation in BPD caregivers. This area appears to have generated multiple theories, but we consider it an emerging literature in respect to application of theories and empirical investigation. Valuable contribution could be made through further micro-analysis and longitudinal observation to determine the prevalence and impact of dissociative phenomena on BPD parents, their experience of caregiving whilst experiencing these phenomena, and the outcomes for their children.

### Literature gaps and limitations: potential pathways forward

There are several gaps identified in this body of literature, the most important being the lack of longitudinal research defining and investigating the role of dissociation in BPD caregivers and their impacts on their children, particularly across infancy. There were no studies identified that provided a robust definition of dissociation in BPD caregivers, grounded in well-articulated theory, and then investigated the impact of caregiver’s dissociation on their children across time. This was unfortunate, given the theoretical importance placed on dissociation as a mechanism of enabling disruptive parenting behaviours. Accurately measuring and understanding the impact of caregiver dissociation on parenting in this population may allow for more specific therapeutic interventions for this population. Another major gap in the literature was father, or family, experiences and impacts on this population; a common theme in the literature where males are underrepresented [79]. None of the sources included focussed on males, partner influences in families, or male parents with BPD. Given that dissociation was posited in some sources as an intersubjective phenomenon, the impact of fathers on the family unit, and their experience of both personal dissociation, and dissociative phenomena in their partners, may allow for further understanding of the dynamics that facilitate or ameliorate intergenerational transmission of trauma, and help to support BPD caregiver’s ability to parent effectively.

Methodologically the main constraints were the quality of the identified studies, and the definition and measurement of dissociation. In relation to the quality of studies, a large portion of the sources identified were theoretical articles. Whilst valuable in adding to the discourse and mapping future directions for study, many of these failed to articulate a consistent definition of dissociation, leading to difficulty in identifying avenues for further study. Similarly in experimental sources, issues related to poor articulation of dissociation, and subsequent difficulty in measuring the construct were common. Explicit focus on, and measurement of, dissociation through observational means would add to the quality of the literature by allowing an understanding of the prevalence of dissociative phenomena in this population and its import as a target for treatment. Finally, a consensus definition of dissociation, and operationalisation of the phenomena in caregiving, would allow future studies to make a more coherent contribution to the field.

### Study limitations and strengths

There were some limitations to our study. Non-English material was excluded due to lack of resources for translation of articles. The focus of the research also precluded comment on dissociation occurring in other disorders (e.g. complex PTSD). Broader clarification may help to understand better the importance in the current population (BPD caregivers and their offspring). A strength and limitation of the thematic analysis is the ability to synthesise and develop themes, but also the inherent subjectivity rooted in this approach. Due to the nature of the sources included and the subject material, there was some difficulty in creating coherent themes. The strength of this approach however was the ability to highly the inconsistency noted in the included sources. The inclusion of varied sources (theoretical, experimental and book chapters) also allowed a broad understanding of area from multiple epistemological perspectives. Considering the relative lack of studies in this area, this was considered an important strength.

## Conclusions

The scoping review suggested that dissociation is a construct of interest in the intergenerational transmission of trauma, possibly impairing caregiving capabilities. However, the concept remains difficult to study and define and the importance of the dissociation in caregivers with BPD is understudied when compared to the theoretical importance granted to the mechanism. Across sources, we identified different ways of conceptualising dissociation, and possibly related parenting behaviours and responses. All of these may benefit from being examined with a focus on dissociation experimentally. Methodological concerns were identified in respect to the difficulty of conceptualising the overlap between concepts – dissociation, BPD, and implications for care of offspring. Particularly hampering empirical investigation was the lack of consistency in theoretical conceptualisation, and subsequent lack of systematised study in the area. There has been increasing focus on parenting as a factor in pathogenesis of psychiatric problems in offspring over the last 40 years, with dissociation a notable and consistent outcome in offspring categorised as ‘disorganised’ in attachment. Nevertheless, there still appears to be a dearth of empirical investigation of caregivers’ experiences in respect to dissociative phenomena when providing care for their infants and the observed effects on their infants. This study potentially offers sufficient integration of current knowledge to provide a platform for further clinical/empirical studies.

### Supplementary Information

**Supplementary Material 1.**

## Data Availability

The datasets used and/or analysed during the current study are available from the corresponding author on reasonable request.
