# Supplementary material for: Dissociation in mothers with borderline personality disorder: a possible mechanism for transmission of intergenerational trauma? A scoping review
Source: Borderline Personal Disord Emot Dysregul. 2024 Mar 11;11:7. doi: 10.1186/s40479-024-00250-7 (PMC10926641; doi:10.1186/s40479-024-00250-7)
Supplement: Supplementary file 1 — Supplementary Material 1. [file 40479_2024_250_MOESM1_ESM.docx]

# Supplementary Materials

### Supplement I: PRISMA Checklist

Preferred Reporting Items for Systematic reviews and Meta-Analyses extension for Scoping Reviews (PRISMA-ScR) Checklist

| **SECTION** | **ITEM** | **PRISMA-ScR CHECKLIST ITEM** | **REPORTED**  **ON PAGE #** |
| --- | --- | --- | --- |
| **TITLE** | | | |
| Title | 1 | Identify the report as a scoping review. | 1 |
| **ABSTRACT** | | | |
| Structured summary | 2 | Provide a structured summary that includes (as applicable): background, objectives, eligibility criteria, sources of evidence, charting methods, results, and  conclusions that relate to the review questions and objectives. | 2 |
| **INTRODUCTION** | | | |
| Rationale | 3 | Describe the rationale for the review in the context of what is already known. Explain why the review  questions/objectives lend themselves to a scoping review approach. | 9 |
| Objectives | 4 | Provide an explicit statement of the questions and objectives being addressed with reference to their key elements (e.g., population or participants, concepts, and context) or other relevant key elements used to  conceptualize the review questions and/or objectives. | 9 |
| **METHODS** | | | |
| Protocol and registration | 5 | Indicate whether a review protocol exists; state if and where it can be accessed (e.g., a Web address); and if available, provide registration information, including the registration number. | 10 |
| Eligibility criteria | 6 | Specify characteristics of the sources of evidence used as eligibility criteria (e.g., years considered, language,  and publication status), and provide a rationale. | 11 |
| Information sources* | 7 | Describe all information sources in the search (e.g., databases with dates of coverage and contact with authors to identify additional sources), as well as the date the most recent search was executed. | 11 |
| Search | 8 | Present the full electronic search strategy for at least 1  database, including any limits used, such that it could be repeated. | Appendix 2 |
| Selection of sources of evidence† | 9 | State the process for selecting sources of evidence (i.e., screening and eligibility) included in the scoping review. | 12 |
| Data charting process‡ | 10 | Describe the methods of charting data from the included sources of evidence (e.g., calibrated forms or forms that have been tested by the team before their use, and whether data charting was done independently or in duplicate) and any processes for obtaining and  confirming data from investigators. | 13 |
| Data items | 11 | List and define all variables for which data were sought and any assumptions and simplifications made. | 13 |
| Critical appraisal of individual sources of evidence§ | 12 | If done, provide a rationale for conducting a critical appraisal of included sources of evidence; describe the  methods used and how this information was used in any data synthesis (if appropriate). | 14 |
| Synthesis of results | 13 | Describe the methods of handling and summarizing the data that were charted. | 24 |

| **SECTION** | **ITEM** | **PRISMA-ScR CHECKLIST ITEM** | **REPORTED**  **ON PAGE #** |
| --- | --- | --- | --- |
| **RESULTS** | | | |
| Selection of sources of evidence | 14 | Give numbers of sources of evidence screened, assessed for eligibility, and included in the review, with  reasons for exclusions at each stage, ideally using a flow diagram. | 13 |
| Characteristics of sources of evidence | 15 | For each source of evidence, present characteristics for which data were charted and provide the citations. | 15-21 |
| Critical appraisal within sources of evidence | 16 | If done, present data on critical appraisal of included sources of evidence (see item 12). | 24 |
| Results of  individual sources of evidence | 17 | For each included source of evidence, present the  relevant data that were charted that relate to the review questions and objectives. | 26 |
| Synthesis of results | 18 | Summarize and/or present the charting results as they relate to the review questions and objectives. | 26-36 |
| **DISCUSSION** | | | |
| Summary of evidence | 19 | Summarize the main results (including an overview of concepts, themes, and types of evidence available), link to the review questions and objectives, and consider the relevance to key groups. | 37-39 |
| Limitations | 20 | Discuss the limitations of the scoping review process. | 39-40 |
| Conclusions | 21 | Provide a general interpretation of the results with respect to the review questions and objectives, as well  as potential implications and/or next steps. | 41 |
| **FUNDING** | | | |
| Funding | 22 | Describe sources of funding for the included sources of evidence, as well as sources of funding for the scoping  review. Describe the role of the funders of the scoping review. | 1 |

*From:* Tricco AC, Lillie E, Zarin W, O'Brien KK, Colquhoun H, Levac D, et al. PRISMA Extension for Scoping Reviews (PRISMAScR): Checklist and Explanation. Ann Intern Med. 2018;169:467–473. [doi: 10.7326/M18-0850.](http://annals.org/aim/fullarticle/2700389/prisma-extension-scoping-reviews-prisma-scr-checklist-explanation)

### Supplement II: Logic Grids for Searches

#### PUBMED

| DISSOCIATION | PARENTING | BORDERLINE PERSONALITY DISORDER |
| --- | --- | --- |
| “dissociative disorders”[mh] OR Dissociative[tiab] OR Dissociate[tiab] OR Dissociat*[tiab] | “FAMILY”[mh] OR “CHILD REARING”[mh] OR Parenting[tiab] OR mother[tiab] OR father[tiab] OR caregiver[tiab] OR care*[tiab] | “BORDERLINE PERSONALITY DISORDER”[mh] OR BPD[tiab] OR Borderline*[tiab] OR Personality Disorder*[tiab] OR Borderline Personality Organisation [tiab] OR BPO[tiab] |

#### PsycINFO

| DISSOCIATION | PARENTING | BORDERLINE PERSONALITY DISORDER |
| --- | --- | --- |
| Dissociation.sh OR exp Dissociative Disorders OR Dissociat*.mp | Exp Family OR exp Parenting OR Parent*.mp OR Parental Absence.sh  OR mother child communication.sh OR mother child relations.sh  OR exp parents OR exp Caregivers OR mother.tw OR father.tw OR parent.tw OR caregiver*.tw OR carer.tw | Borderline Personality Disorder.sh OR Borderline States.sh OR Complex PTSD.sh OR BPD.tw OR Borderline*.tw OR Personality Disorder.tw OR Borderline Personality Organi?ation.tw |

#### PsycEXTRA

| DISSOCIATION | PARENTING | BORDERLINE PERSONALITY DISORDER |
| --- | --- | --- |
| Dissociation.sh OR exp Dissociative Disorders OR Dissociat*.mp | Exp Family OR exp Parenting OR Parent*.mp OR Parental Absence.sh  OR mother child communication.sh OR mother child relations.sh  OR exp parents OR exp Caregivers OR mother.tw OR father.tw OR parent.tw OR caregiver*.tw OR carer.tw | Borderline Personality Disorder.sh OR Borderline States.sh OR Complex PTSD.sh OR BPD.tw OR Borderline*.tw OR Personality Disorder.tw OR Borderline Personality Organi?ation.tw |

#### PubMed PMC

| DISSOCIATION | PARENTING | BORDERLINE PERSONALITY DISORDER |
| --- | --- | --- |
| “dissociative disorders”[mh] OR Dissociative[tw] OR Dissociate[tw] OR Dissociation[tw] OR negative dissociation[tw] OR positive dissociation[tw] OR structural dissociation[tw] OR depersonalisation[tw] OR derealisation[tw] OR depersonalization [tw] OR derealization [tw] | “FAMILY”[mh] OR “CHILD REARING”[mh] OR Parenting[tw] OR mother[tw] OR father[tw] OR caregiver[tw] OR carer [tw] | “BORDERLINE PERSONALITY DISORDER”[mh] OR BPD[tw] OR Personality Disorder*[tw] OR Borderline Personality Organisation [tw] OR BPO[tw] |

#### PsycARTICLES

| DISSOCIATION | PARENTING | BORDERLINE PERSONALITY DISORDER |
| --- | --- | --- |
| Dissociation OR Dissociative Disorders OR Dissociat*.mp | Family OR Parenting OR Parent*.mp OR Parental Absence  OR mother child communication OR mother child relations  OR parents OR Caregivers OR mother.tw OR father.tw OR parent.tw OR caregiver*.tw OR carer.tw | Borderline Personality Disorder OR Borderline States OR Complex PTSD OR BPD.tw OR Borderline*.tw OR Personality Disorder.tw OR Borderline Personality Organi?ation.tw |

#### Embase

| DISSOCIATION | PARENTING | BORDERLINE PERSONALITY DISORDER |
| --- | --- | --- |
| Dissociation/exp OR Dissociative Disorders/exp OR “Dissociat*”:ti,ab | Family/exp OR child parent relation/exp OR “child parent relationship”:ti,ab OR “child parent spatial pattern”:ti,ab OR “correlation, parent child”:ti,ab OR “parent child correlation”:ti,ab OR “parent child relation”:ti,ab OR “parent child relationship”:ti,ab OR “parent infant bonding”:ti,ab OR “parent infant relation”:ti,ab OR “parent-child relations”:ti,ab OR “parental role”:ti,ab OR “parenting”:ti,ab | borderline state/exp OR “borderline”:ti,ab OR “borderline personality”:ti,ab OR “borderline personality disorder”:ti,ab OR “borderline psychosis”:ti,ab OR “personality disorder, borderline”:ti,ab |

#### CINAHL

| DISSOCIATION | PARENTING | BORDERLINE PERSONALITY DISORDER |
| --- | --- | --- |
| MH “Dissociative Disorders +” OR TX “Dissociat*” | MH “Family +” OR TX “Mother Infant” OR TX “Father Infant” OR MH “Caregiver” | MH “Borderline Personality Disorder” OR TX “Complex PTSD” OR TX “BPD” or TX “Borderline Personality” |

#### Scopus

| DISSOCIATION | PARENTING | BORDERLINE PERSONALITY DISORDER |
| --- | --- | --- |
| “Dissociat*” | “Family” OR “Child Rearing” OR “Parenting” OR “Mother*” OR “Father*” OR “Care*” | “Borderline Personality *” OR “BPD” OR “BPO” |

#### Web of Science

| DISSOCIATION | PARENTING | BORDERLINE PERSONALITY DISORDER |
| --- | --- | --- |
| “Dissociat*” | “Family “OR “Child Rearing” OR “Parenting” OR “Mother*” OR “Father*” OR “Care*” | “Borderline Personality *” OR “BPD” OR “Borderline Personality Disorder” OR “Borderline*” OR “Personality Disorder*” OR “Borderline Personality Organisation” OR “BPO” |

#### PTSDpubs

| DISSOCIATION | PARENTING | BORDERLINE PERSONALITY DISORDER |
| --- | --- | --- |
| “Dissociation Assessment Instruments” OR MAINSUBJECT.EXACT.EXPLODE("Depersonalization Disorder") OR MAINSUBJECT.EXACT.EXPLODE("Dissociative Amnesia") OR MAINSUBJECT.EXACT.EXPLODE("Dissociative Identity Disorder") OR MAINSUBJECT.EXACT.EXPLODE("Dissociative Fugue") OR MAINSUBJECT.EXACT.EXPLODE("Hallucinations") OR “dissociat*” | MAINSUBJECT.EXACT.EXPLODE("Parents") OR “Parenting” OR MAINSUBJECT.EXACT.EXPLODE("Interpersonal Interaction") OR “Mother” OR “Father” OR “Caregiving” | MAINSUBJECT.EXACT.EXPLODE("Borderline Personality Disorder") OR “BPD” OR “Borderline Personality” |

### Supplement III: Full Descriptive Table

| **1st Author (Year), Location, Type** | **Design** | **Aims** | **Population** | **Methods** | **Findings related to dissociation and BPD carers** | **Theory of Dissociation** | **What work has been done to advance understanding of dissociative caregiving in this population?** |
| --- | --- | --- | --- | --- | --- | --- | --- |
| Blizard (2003), USA, Journal Article | Non-systematic review and opinion | Not explicitly stated. To present a theoretical model of disorganized attachment and dissociation, and a treatment model based on findings. | N/A | Theoretical discussion and synthesis and presentation of case material | -Theoretical discussion  - Dissociation mechanism for trauma transmission due to ‘double bind’ situation where child is subject to threat from caregiver  - BPD not specifically addressed but dissociation is implied as the mechanism for development of BPD | Proposes dissociation as a mechanism for disrupted caregiving (mother) and as a way to deal with ‘double-bind’ situations whereby the child is subject to seeing its caregiver as hostile or aggressive | Theoretical discussion with explicit theoretical basis |
| Crandell (2003), UK, Journal Article | Non-randomised experimental study | To investigate mother-infant relations when mothers have borderline personality disorder | Mothers with borderline personality disorder + their infants; control group of mothers with no history of disorder + their infants. | No dissociation measure. Videotape analysis and rating by blinded rater based on Murray et al (1996) Criteria. Measured Pre- and Post-still-face procedure, and during face-to-face play post-still-face. | -Focus on BPD Caregivers suggest heightened insensitive / intrusive parenting from mothers (BPD group scored lower on non-intrusive sensitivity) also significantly less sensitive and more intrusive  -Implicitly, dissociation can be seen as the mechanism enabling intrusive insensitivity, and impairing ability to be sensitive | Dissociation is implied or explicitly addressed, but no definition is offered for the construct by the authors | Observation of behaviour with implicit idea |
| Haltigan (2019), Canada, Journal Article | Latent trait modelling analysis of AMBIANCE Measure (Factor Analysis) | To apply the Item Response Theory (IRT) modelling techniques to a large sample of data from the Atypical Maternal Behaviour Instrument for Assessment and Clarification (AMBIANCE) in order to analyse the latent traits in the instrument, with a view to identification of the most efficient items that allow for maximal information in minimal time in a clinical setting. | Item-level AMBIANCE data; 6 subsamples (pooled n = 343) from various parent studies conducted in western countries. | Item level data on the AMBIANCE measure pooled; transformed to binary scale and analysed. | -Dissociative behaviour subdimension 3B highlights three behaviours of concern; "Exhibits sudden change in mood unrelated to environment", "Exhibits sudden loss of affect" and "Treats inanimate objects as animate".  -Exhibits sudden change in mood unrelated to environment occurred infrequently (.02 proportionally) but exhibited significant severity in terms of impact on infant (6.33 severity). Exhibits sudden loss of affect and treats inanimate objects as animate were less in severity of impact (0.73 and 2.69 respectively).  - Dissociation subsumed under fearful.. disoriented behaviour | Dissociation is implied or explicitly addressed, but no definition is offered for the construct by the authors | Observation of behaviour with explicit focus or mention |
| Hesse (2000), USA, Journal Article | Non-systematic review and opinion | Provide a descriptive account of processes that identify disorganized attachment status across lifespan | Presentation of cases and summary synthesis of literature regarding disorganised attachment | Presentation and synthesis of ideas relating to attachment, disorganised attachment and dissociation | - Unresolved / disorganised attachment linked to borderline personality disorder  - Dissociative behaviour in parents a second generation effect due to unresolved mental states in their own caregivers  - Dissociative parental behaviour are frightening and disorganising for infants | Dissociation is seen as a symptom of BPD but not necessarily discussed as a causative agent of mother-child disruption | Observation of behaviour with explicit focus or mention; Theoretical discussion where there is no explicated model |
| Hobson (2005), UK, Journal Article | Non-randomised experimental study | Assessment of interactional quality and attachment of 12 month old infants of mothers with BPD; evaluation of maternal intrusive insensitivity. | Mother infant Dyads with 12-month old infants. 10 Mothers with BPD; 22 control mothers without psychopathology | Modified set situation was applied to each mother infant dyad. Still face phase (90seconds). Rapprochement and spatula phase then administered. Rating of whole situation then applied. | -Dissociation may be associated with disorganised attachment. Infants experience behavioural dissociation in relation to mothers in strange situation as a reflection of "dissociated states of mind"  -Interpersonal relations between mothers with BPD and infants are posited to have a unique quality of maternal intrusiveness and insensitivity characterised by maternal changes in mood and abrupt switches in internal states  -Authors do not explicitly describe this as dissociative | Dissociation is implied or explicitly addressed, but no definition is offered for the construct by the authors | Observation of behaviour with implicit idea |
| Hobson (2009), UK, Journal Article | Non-randomised experimental study | To assess how women with borderline personality disorder engage with their 12 to 18-month-old infants in separation– reunion episodes. | Mother infant Dyads. Cohort 1: 12-month old infants. 10 Mothers with BPD; 22 control mothers without psychopathology (n=32). Cohort 2: 27 mother-infant dyads, where the mothers' had BPD. | Modified set situation was applied to each mother infant dyad. Still face phase (90seconds). Rapprochement and spatula phase then administered. Rating of whole situation then applied. | -BPD mothers distinguished by the presence of Frightened / disoriented behaviour (AMBIANCE), under which dissociative behaviour is a subcategory  - Intergenerational trauma from BPD mothers to infants is seen as due to disruptions in affective communication with dissociation as a mechanism of unclear importance | Dissociation is implied or explicitly addressed, but no definition is offered for the construct by the authors | Administration of validated measure |
| Hulette (2011), USA, Journal Article | Cohort study | Investigate the intergenerational relationships between trauma and dissociation. | 67 mothers and their children, aged 7-8 years old. 36 boys, 31 girls. | Trauma histories of parents and children were assessed using: Brief Betrayal Trauma Survey (BBTS) and Brief Betrayal Trauma Survey - Parent report (BBTS - Parent). Categorised based on responses to 'high betrayal trauma', 'low betrayal trauma' or 'no betrayal trauma' conditions. Parents dissociation was assessed via administration of the Dissociative Experiences Scale (DES). Children's dissociation was assessed by the administration of the Child Dissociative Checklist (CDC). Data was analysed using ANOVAs, Chi Square test and independent sample t-tests, as well as post-hoc testing for the ANOVAs. | - Dissociation is posited as a logical extension of betrayal trauma, and further revictimization (dissociation as defensive reaction to trauma)  -dissociation is a mechanism for intergenerational traumatisation of children through impaired awareness of interpersonal threats in dissociative caregivers | Dissociation is defined broadly as any interruption to *subjective* integration of various mental systems (behavioural, emotional, sensory etc.), which may lead to lapses in effective parenting AND Dissociation most likely to occur in those who have a close relationship to the perpetrator. In mothers who have BPD or suffer high betrayal trauma and dissociation, the theory states that the awareness of external threats to their offspring may be diminished due to overreliance on defensive dissociation to deal with stressful or affectively salient stimuli and situations | Administration of validated measure |
| Kiel (2011), USA, Journal Article | Non-randomised experimental study | investigate the association between borderline personality pathology and at-risk parenting. Investigation the nature of parenting in response to infant distress in mothers with and without borderline personality pathology. | 99 infants and their mothers; divided into ''high borderline personality" and "low borderline personality groups". Infants range from 12-23 months. | Mothers screened with Borderline Evaluation of Severity over Time (BEST); Difficulties in emotion regulation scale (DERS); Depression, Anxiety, Stress Scales (DASS-21); 1180s of reunion in the "Strange situation" was coded. Infant affect, maternal affect, maternal behaviour were all scored. Demographic testing revealed no significant differences between high BP and low BP conditions.  Analysis consisted of examining contingencies between infant distress and maternal affective and behavioural responses. Contingencies (maternal response) was lagged 2 seconds behind infant state. Infant state coded second by second as distress vs. non-distress. Maternal response coded affect positive, negative or neutral; behaviour as comforting, distraction, insensitive and neutral. Chi square values were derived to predict expectant contingencies in each group, and in each dyad. Sub analyses were carried out including assessing frequency of maternal responses to infant distress, as well as latency of response to infant distress between groups and changes in probability of responses across duration of infant distress. Infant response to maternal response (transactional response) was also calculated through repeated measures ANOVA. | - Differences consisted of more insensitive maternal responding as infant distress persisted across time (measured at 5s latency) in the High BP group. Mothers in high BP group are less likely to respond with positive affect to their infants distress as compared to low-bp group. Insensitive behaviour then predicted a rise in infant distress.  -Dissociation is not taken as an explanatory hypothesis or explicitly assessed. The nature of the study is to investigate transactions in the mother-infant relationship, specifically the response to infant distress, in those with High-BP.  Authors note that frightened / frightening and confusing behaviours were not assessed.  Also infant distress magnitude and changes in this were not assessed, nor was change in response in maternal behaviour contingent on infant distress magnitude. | Dissociation is characterised as a lack of integration of thoughts, emotion, identity and / or physical sensations into the stream of consciousness (APA 2013). Theory presented is not explored further than this definition, expect to suggest that parents serve as models for their children such that "... dissociative tendencies may increase the likelihood children develop similar behavioral patterns (Bariola, Gullone, & Hughes, 2011)." and further that "...Children's dissociative behaviors develop in order to cope (Nugent, Sledjeski, Christopher, & Dielahanty, 2011)." | Administration of validated measure |
| Lewis (2020), USA, Journal Article | Non-randomised experimental study | Examining parent factors related to changes in dissociation symptoms in childhood. | 68 Mothers (‘likely BPD’ and other) and their preschool aged children. | Dyads completed assessment two years apart (T1 and T2). 2.5h assessment session. Mothers completed a battery of self report questionnaires about their and their children’s mental health symptoms. Mothers administered the following:  Difficulties in Emotion Regulation Scale administered at T1 and T2 (DERS; Gratz & Roemer, 2004); Dissociative Experiences Scale administered at T2 only (DES; Bernstein & Putnam, 1986).   Child dissociation was assessed using a modified subscale for the Child Behaviour Checklist (CBCL; Achenbach & Rescorla, 2000) at T1 and T2. | - Maternal dissociation (only assessed at T2) was moderately correlated with child dissociative behaviours at T2 (r(36) = .62,p< .001) and at T1 (r(34) = .45,p= .007). Maternal emotion dysregulation at T2 was moderately associated with maternal dissociation (r(36) = .58,p< . 001). T1 dissociation not measured so examination between maternal dissociation and children’s dissociative or disrupted behaviours not assessed.  -Maternal dissociation associated with dissociation in children during the early school years  -Child dissociation associated with risk as there is was a positive correlation between problem behaviours and child dissociation.  -mother emotion dysregulation and dissociation was a factor associated with children’s dissociative symptoms | Dissociation is defined broadly as any interruption to *subjective* integration of various mental systems (behavioural, emotional, sensory etc.), which may lead to lapses in effective parenting | Theoretical discussion with explicit theoretical basis |
| Liotti (2004), Italy, Journal Article | Non-systematic review and opinion | Familiarise the reader with findings suggesting that disorganised attachment is central to trauma-related disorders, and the propensity to react to traumatic events with dissociation is related to disorganisation of early attachment. | N/A | Summary and synthesis of evidence and theoretical opinion | -BPD a form of clinically distinct disorder linked to early attachment disorganisation  - Attachment disorganisation linked to failure of integration of mutually contradictory internal working models  - Interpersonal trauma or 'relational trauma' are conceptualised as manifestations of interrupted relationships between mother and infant. Dissociative phenomena are again seen as the phenomenological manifestation of "unresolved" trauma responses whereby there is an intrusion, conscious or unconscious, of traumatic thoughts / memories / affects / sensations, into the caregivers mind. Thus dissociation is a manifestation of trauma response, and also a causative mechanism for 'passing on trauma' | Proposes dissociation is in itself a painful experience that occurs interpersonally rather than intrapsychically and disrupts caregiving | Theoretical discussion with explicit theoretical basis |
| Liotti (2011), Italy, Book Chapter | Non-systematic review and opinion | Book Chapter. Discussion of the relationship between attachment and dissociation. | N/A | Summary and synthesis of evidence and theoretical opinion | - Dissociation as key mechanism which is brought about by simultaneous activation of attachment system with other biobehavioural systems where caregiver response is not adequate to sooth (disorganised attachment)  - The theory presented departs from other models of dissociation as (intrapsychic) defence against traumatic memory / experience. Instead, dissociation and dissociative experiences are themselves painful, and are brought about by conflictual intersubjective "Internal Working Models" i.e. ways of being with others that are encoded in implicit memory. The theory here hinges on the idea that when the attachment biobehavioural system is activated (such as when providing care for your infant / child), this can trigger activation of unresolved ways of being with other (IWMs) which may contain material which is conflictual, leading to inability to provide adequate care. | Proposes dissociation is in itself a painful experience that occurs interpersonally rather than intrapsychically and disrupts caregiving | Theoretical discussion where there is no explicated model |
| Lyons-Ruth (2012), USA, Commentary Article | Non-systematic review and opinion | Commentary article on Stepp, Whalen, Pilkonis, Hipwell, and Levine (2011) | N/A | Response article | -Suggests that in BPD caregivers (and other) more work needs to be done to understand if there are "specific kinds of disruption in early communication (i.e. frightening vs. dissociative vs. role-confused) that are particularly characteristic of parents with different kinds of disorders  -creating disorder-specific phenotyping of maternal behaviours is seen as important to target interventions | Dissociation is implied or explicitly addressed, but no definition is offered for the construct by the authors | Observation of behaviour with implicit idea; Theoretical discussion where there is no explicated model |
| Macfie (2014), Spain, Journal Article | Non-systematic review and opinion | Review of evidence whether BPD has its origins in part due to failure to negotiate early childhood tasks, focusing on the role of parenting. | N/A | Summary and synthesis of evidence and theoretical opinion; review of evidence for role of parenting in aetiology of BPD | -Dissociation only implied in the article  - A longitudinal pathway is explicated (Fig19.1) whereby infant disorganisation is seen as a step on a pathway through development to BPD in adolescence / early adulthood. "Parent representations of childhood attachment" are seen as a significant factor for parenting and impact on the relational factors at play in the child's development of psychopathology. | Dissociation is implied or explicitly addressed, but no definition is offered for the construct by the authors | Theoretical discussion with explicit theoretical basis |
| Mosquera (2014), Spain, Journal Article | Non-systematic review and opinion | Explicate pathway(s) from attachment disruption to specific symptomatology in BPD patients. | N/A | Summary and synthesis of evidence and theoretical opinion; integration of the Theory of Structural Dissociation of the Personality with mother-infant attachment in BPD context | -Theory of Structural Dissociation of the Personality (TDSP); "The word dissociation is used to describe a mechanism involved in a fundamental division within the personality" p.2.  -The model relates to 'dissociation' as above occurring in relation to simultaneous activation of conflicting motivational subsystems which occurs chronically in relation to attachment figures in infancy / childhood. Fundamental division is further described under TDSP as 'parts' labelled 'apparently normal personality (ANP)' and 'emotional personality (EP)'. Each of these 'parts' operates in varying degrees of 'dissociation' to one another, where more serious dissociation leads to 'ego-dystonic' experiences.  -BPD or a subtype of BPD is seen as fundamentally dissociative and impacting | Proposes that dissociation happens intrapsychicially and splinters the personality in such a way that it become impossible to access certain ‘parts’ of the personality, usually divided into ‘emotional’ and ‘apparently normal’ parts, leading to disrupted caregiving in those who have dissociated ‘parts’ of the personality | Theoretical discussion with explicit theoretical basis |
| Mucci (2021), Italy, Journal Article | Non-systematic review and opinion | To clarify the effects of intergenerational psychological traumatization, stratifying into two levels; 1) human agency (lack of attunement) and 2) actual abuse, maltreatment or incest "as seen in borderline psychopathology" | N/A | Summary and synthesis of evidence and theoretical opinion | -Dissociation is slightly differently interpreted than other studies in that the dissociation is a defence against an unregulated hypo- or hyper-aroused state i.e. a metabolically and biologically dangerous situation. This comes with a consequence of 'encoding' in a preverbal (non-hippocampal) area of the 'memory trace' of the event, including relational aspects that are stored as 'triggers'.  -Dissociation is seen as a response that causes or is fundamentally entangled with 'splits in the personality'. Splits in the personality fundamentally related to internal working models or dyads that are internalised; victim / persecutor. These splits are fundamentally at the root of borderline pathology. Borderline pathology is transmitted to future generations thorough reactivation of the split parts of the personality / dissociative mechanisms due to activation of atttachment system through caring behaviours (or not activating the system?). | Dissociation is located as a right-brain-hemispheric phenomena that occurs in respect to ‘relational’ trauma of a pre-oedipal kind (i.e. developmentally early). Dissociation is seen as an intrapsychic defence against overwhelming emotions that are triggered in infants in response to human-inflicted trauma. Dissociation then operates unconsciously and pre-verbally, and is associated with internalisation of split-off ‘victim-persecutor’ internal working models, which then govern emotional response to others including in a caregiving setting i.e. in BPD adults providing care for offspring | Administration of validated measure |
| Ozturk (2006), Turkey, Journal Article | Cohort study | To assess the dissociative experiences, including borderline personality disorder, among first degree relatives of dissociative patients. | 24 Dissociative patients (18 diagnosed with DID; six with DDNOS). Nineteen female, 24.2 years mean age. 50 family members, first degree relatives, of these patients were contacted. 20 Mothers, 10 fathers, 20 siblings. 27 were female, mean age 41.1, | Dissociative expriences scale (DES-II; Brenstein & Putnam, 1986; Carlson & Putnam, 1993),  Structured Clinical Interview for DSM-III-IV Personality Disorders (SCID-II) Borderline Personality Disorder Section - Turkish version (Coskunol, Bagdiken, Sorias, & Saygili, 1994)  Childhood Trauma Questionnaire (CTQ) Bernstein et al., 1994)  Three (3) family members who had a DES score >25 were administered the Structured Clinical Interview for DSM-IV Dissociative Disorders (SCID-D; Steinberg 1994)  It is unclear what statistical analyses were applied as they are not stated in the method or results sections, however data is presented in tabular format. | -Family members "reported more frequent mood fluctuations, intense anger, transient dissociative experiences or paranoid ideas, and identity confusion more frequently than controls " pp293  -Unclear what statistics are being reported  - T scores are however reported and there appears to be significance at various levels in between group differences for the symptoms listed above AND for total number of borderline personality disorder criteria (t+2.75, p<.01) between dissociative families and non-dissociative families  -Physical neglect on the CTQ is most closely correlated to total number of borderline criteria at r=.056, p<.001. Physical neglect can be seen as a logical extension to an unavailable caregiver who is dissociative, however the study design does not effectively allow for this conclusion to be drawn as we don't know what relation specifically the family member is to the dissociative patient however we do know that 30 of the family members were mother or father (60%) and the remaining 40% were siblings.  -So it is likely that parents and siblings of dissociative individuals experience physical neglect and exhibit borderline personality disorder traits at a rate higher than a control population. The direction and nature of the interaction isn't clear despite the authors hypotheses. | Dissociation is defined broadly as any interruption to *subjective* integration of various mental systems (behavioural, emotional, sensory etc.), which may lead to lapses in effective parenting | Administration of validated measure |
| Reinelt (2014), Germany | Cohort study | To test longitudinally and in a community-based sample if maladaptive mother-child interactions (conceptualized by an insensitive parenting style and discrepancies in the perception of psychopathological problems of the offspring) mediate the relationship between maternal borderline symptomatology and BPD symptoms of the offspring about 5 years later. (p.11) | 230 families comprising 295 Children and their biological mothers; all families involved in the Griefswald Family Study. | Assessed with instruments at two timepoints; initial time T0 and T1 (approx.. 5 years on).   Maternal BPD symptoms assessed with the self-rating part of the German Version of the SCID for DSM-III-R (SCID-II)  Perceived insensitive habitual parenting style of the mothers, adolescents completed the EMBU ('own memories concerning upbringing') scale. Self report questionnaire consisting of three scales (rejection, emotional warmth and overprotection). 4 point Likert scale.  Primary caregiver filled out CBCL and offspring filled out he corresponding Youth Self Report (YSR). 96 congruent items in CBCL and YSR were used to calculate discrepancy scores. CBCL scores subtracted from YSR scores separately for internalizing and externalizing disorders, and these scores were used for further analysis.  t1 assessments:  BPD symptoms of adolescents / young adults were examined using the German version of the SCID-II interview for DSM-IV.  Data was analysed in SPSS; Path analyses were conducted using robust maximum likelihood SEM procedure with AMOS 18; gender controlled. | -Mothers with high BPD symptoms tend to ignore or be unaware of (high discrepancy) internalizing problems in their children; possible explanatory hypothesis of mothers dissociating from these emotions and issues in themselves and their offspring threatening to reactivate these issues. | Dissociation is seen as a symptom of BPD but not necessarily discussed as a causative agent of mother-child disruption | Theoretical discussion with explicit theoretical basis |
| Schore (2001), USA | Non-systematic review and opinion | To describe the negative impact of traumatic attachment on neuro-development and infant mental health, summarise the neurobiology of infant trauma and the neuropsychology of disorganised / disoriented attachment. | N/A | Overview of the neurobiological consequences of early relational trauma on brain development, affect regulation and infant mental health including dissociative response and issues | - BPD Caregivers may be predisposed to dissociation  - Dissociation is an interpersonal defense, and also an intrapersonal defence  - The dissociative defense has its basis in primitive, right hemispheric neurobiology  - Early dissociation can impair formation of personality and impair ability to engage in ‘right-hemisphere to right-hemisphere’ communication that is important in dyadic co-regulation of affects, as seen in caregiving | Dissociation is discussed in terms of a neurobiological defence against metabolic dysregulation occurring as a part of stress cascade responses in the face of unregulated emotional activation. The neurobiology of dissociation in infants is discussed, as well as the interpersonal consequences of dissociation and the long term effects of (infant) dissociation. Maternal dissociation is an automatic blunting response, primed from infancy, and disrupts infant attachment through ‘suboptimal neurobiological priming’ i.e. Mothers are unable to effectively regulate their own offspring due to their own dissociative responses | Theoretical discussion where there is no explicated model |
| Stepp (2011), USA | Non-systematic review and opinion | To describe the parenting strategies that might explain transmission of intergenerational trauma from mothers with BPD to their offspring. | N/A | Summary and synthesis of evidence and theoretical opinion; parenting strategies (behavioural) and their relation to transmission of trauma from BPD mothers to their offspring | -BPD parenting characteristic in the oscillations from over- involvement to under-involvement | Dissociation is implied or explicitly addressed, but no definition is offered for the construct by the authors | Theoretical discussion where there is no explicated model |
| Zalewski (2014), USA | Cohort study | To examine the associations between Maternal BPD and parenting of 14-17yo girls, and their mothers through assessment of several different cohorts longitudinally | Girls from the Pittsburgh Girls Study (PGS, N=2,451). Urban community, girls aged 15-17; biological mothers and their adolescent daughters n=1,598. Data from 3 cohorts (aged 5, 6, 7 at first data collection) of girls and their mothers from a community sample. Data collected at waves 9, 10 and 11 representing the ages 15, 16 and 17 of the girls, and their mothers. Biological mothers only sampled | Tests administered at 1 year intervals. Participants recruited at age 5, 6 and 7. Each mother-daughter dyad followed up for 12 years. In home interviews conducted separately by trained interviewers. Mothers reported on own psychopathology and their daughters' temperaments. Daughters reports on their parents' use of psychological and behavioural control.  Measures include:   Three subscales from the 18-item child report of the the Parent Behaviour Inventory (Schludermann & Schludermann, 1971). Three subscales were intrusiveness, control through guilt and acceptance of individuation.  Behavioural control assessed via parent report on the Conflict Tactics Scale: Parent-Child Version (Straus, Hamby, Finkelhor, Moore & Runyan, 1998).   Maternal BPD Symptoms using 9 item self report The International Personality Disorders Examination (Loranger, Sartorius, Andreoli, & Berger, 1994).  Negative emotionality measured by parent report when girls were age 15; used emotionality, activity and sociability temperament survey (Buss & Plomin, 1984).  Parents reported on girls lack of self control as measured through the 10 item self control subscale of the social skills rating system (elliott, gresham, freeman, & McCloskey, 1988).  Maternal depression measured by the BDI-II (Beck, steer & Brown, 1996). Maternal Alcohol Use Severity measured each year using the Alcohol Use Disorder Identifcation Test (Barbor, De la Feunte, Saunders & Grant, 1992). | -Tests impact of BPD symptoms on adolescent girls behaviour.  - One of the three factor model of BPD symptoms presented (Based on theoretical review by Trull et al., 2010) is 'identity disturbance', which includes emptiness, depersonalization and changing feelings about the self.  - Identity disturbance was related to negative parenting characteristics when in a three factor model. When all factors were entered into the model simultaneously, however, only affective / behaviourally dysregulated component of BPD was related to parenting variables.  -Dissociation not specifically focussed on but identity disturbance not related to BPD parenting as well as affective / behavioural dysregulation - Recommended observational studies of dynamics between BPD-offspring to better understand nature of disruptive dynamics | Dissociation is implied or explicitly addressed, but no definition is offered for the construct by the authors | Theoretical discussion where there is no explicated model |
